# RNA structure-wide discovery of functional interactions with multiplexed RNA motif library

**DOI:** 10.1038/s41467-020-19699-5

**Published:** 2020-12-08

**Authors:** Kaoru R. Komatsu, Toshiki Taya, Sora Matsumoto, Emi Miyashita, Shunnichi Kashida, Hirohide Saito

**Affiliations:** 1 Department of Life Science Frontiers, Center for iPS Cell Research and Application, Kyoto University, 53 Kawahara-Cho, Shogoin, Sakyo-Ku, Kyoto, 606-8507 Japan; 2 Twist Bioscience, 681 Gateway Blvd South, San Francisco, CA 94080 USA

**Keywords:** RNA, High-throughput screening, RNA, Transcriptomics

## Abstract

Biochemical assays and computational analyses have discovered RNA structures throughout various transcripts. However, the roles of these structures are mostly unknown. Here we develop folded RNA element profiling with structure library (FOREST), a multiplexed affinity assay system to identify functional interactions from transcriptome-wide RNA structure datasets. We generate an RNA structure library by extracting validated or predicted RNA motifs from gene-annotated RNA regions. The RNA structure library with an affinity enrichment assay allows for the comprehensive identification of target-binding RNA sequences and structures in a high-throughput manner. As a proof-of-concept, FOREST discovers multiple RNA-protein interaction networks with quantitative scores, including translational regulatory elements that function in living cells. Moreover, FOREST reveals different binding landscapes of RNA G-quadruplex (rG4) structures-binding proteins and discovers rG4 structures in the terminal loops of precursor microRNAs. Overall, FOREST serves as a versatile platform to investigate RNA structure-function relationships on a large scale.

## Abstract (web summary)

Structured RNA motifs can be obtained by structure probing, duplex capture, and motif prediction. Here the authors develop a multiplexed affinity assay system to identify functional protein interactors from an RNA structure library with validated or predicted RNA motifs.

## Introduction

Functional RNA elements have specific RNA sequences and structures that work together as a functional unit. Discovering RNA structures from a wide variety of transcripts is a critical step in finding functional RNA elements and understanding their regulatory mechanisms. From the perspective of bioinformatics, homology-based searching for both conserved sequences and secondary structures is effective at finding structured RNA motifs^1–4^. Structure probing and prediction software have elucidated transcriptome-wide RNA structural architectures, called the RNA structurome, and revealed numerous RNA structures^5,6^. Transcriptome-wide double-stranded RNA mapping is also critical for identifying higher-order architectures^7–10^. These strategies to determine RNA structures provide the means to investigate the regulatory networks governed by functional RNA elements.

Although RNA structures have been identified, their roles remain mostly unknown. Datasets of RNA structures have been analyzed in combination with large-scale analyses using deep sequencing to elucidate biological phenomenon involving RNA structures^11^. However, transcriptome-wide analysis with sequencing (e.g., CLIP-seq) has only revealed footprints of targeted RNA sequences, which are not equal to RNA elements. Thus, subsequent experiments are needed to determine the structural boundaries of continuous RNAs for the discovery of the RNA elements. Furthermore, current sequencing-based methods suffer from technical limitations regarding transcribed RNAs in specific cells and structure-dependent amplification biases in the library preparation.

**Fig. 1:**
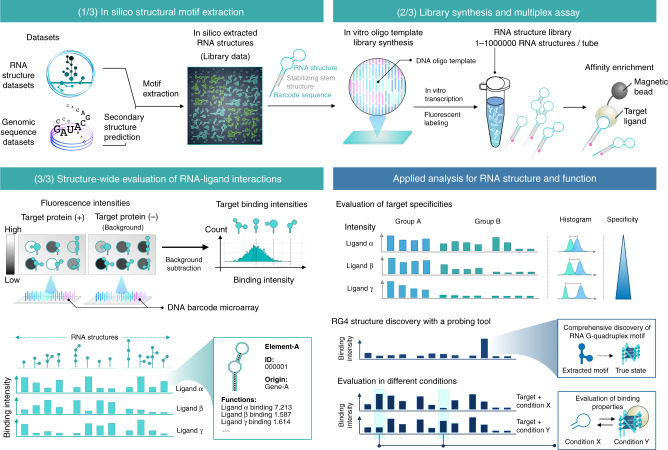
Overview of FOREST. FOREST identifies functional and structured RNA motifs on a large scale. It consists of three procedures. (1) In silico RNA motif extraction. Target RNA motifs are systematically extracted from the structural RNA datasets. The definition of the target motif is flexibly determined. See also Fig. 2, Supplementary Figs. 1–4, and Methods. Extracted RNAs are conjugated to the stabilizing stem structure and RNA barcodes for massively parallel quantification of each RNA in the library. (2) After adding the T7 promoter sequence, reverse complementary DNA sequences are synthesized as an oligo pool. FOREST can quantify the function of all RNA structures in the library in combination with a biochemical assay (e.g., profiling of RNP interactions by RNA-affinity enrichment assay). (3) Analysis for structure-wide discovery of a functional RNA element. To quantify the abundance of the RNA probes, we use a DNA barcode microarray that captures complementary RNA barcodes via specific RNA–DNA hybridization. The output of massive quantification provides the functional score of each RNA, enabling the comprehensive identification of functional RNA-ligand interactions. The results of FOREST can be applied to further analyses, including the evaluation of binding properties at different conditions and the identification of structured RNAs (e.g., RNA G-quadruplexes) (right bottom).

To overcome these limitations, we aim to develop a method for the RNA structure-wide discovery of functional interactions for target ligands. FOREST (folded RNA element profiling with structure library) is a system that quantitatively analyzes interactions using a large number of structured RNA motifs extracted from natural RNAs revealed by structure probing, duplex capture, and motif prediction (Fig. 1). FOREST utilizes RNA structure libraries that were designed by in silico RNA motif-extraction and transcribed from in vitro oligo pools. Hence, FOREST can evaluate a variety of RNA structures regardless of the organism, genome, and transcriptional levels. Furthermore, using a DNA barcode microarray and RNA–DNA hybridization in a quantification step, FOREST can eliminate reverse transcription (RT) and polymerase chain reaction (PCR) amplification bias from the results. This bias-free quantification can directly evaluate the interactions of highly structured RNA such as RNA G-quadruplex (rG4), a noncanonical RNA structure that is still challenging to detect with current sequencing-based methods.

In this study, we implement FOREST to profile RNA–protein (RNP) interactions and demonstrate its potential applications. We first design an RNA structure library that includes 1800 human precursor microRNA (pre-miRNA) loops and structured RNA controls (e.g., rG4). We then profile RNP interactions between the pre-miRNA loop sublibrary and various RNA-binding proteins (RBPs) to measure the protein-binding intensities of each RNA structure, uncovering both essential RNA sequences and structured motifs for the interactions. Second, we apply FOREST to characterize the structure-binding preferences of RBPs by utilizing affinity-based enrichment. We reveal different binding preferences of rG4-targeting RBPs and identify rG4 structures from the pre-miRNA loops. Finally, we design another RNA structure library by extracting structures from human 5′UTR and the HIV-1 RNA genome to analyze the RNP interactions of a cellular EIF3 complex. We identify EIF3-complex binding elements that regulate translation in living cells.

## Results

### Development of FOREST

**Fig. 2:**
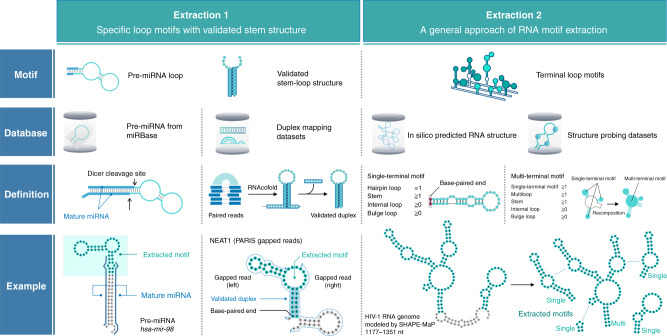
Structural motif extraction for the RNA structure library. Motif extraction obtains RNA structures from datasets to design the RNA structure library in FOREST. Extraction 1: A method to extract a validated stem–loop structure. In this study, pre-miRNA, a region surrounded by mature miRNAs, was recognized as a validated stem-loop motif, and the loop region with a part of the stem was extracted from human pre-miRNAs registered in miRBase. The Dicer cleavage site determined the boundary of the extracted pre-miRNA loop motif. We also extracted experimentally validated RNA stems from the data set of the high-throughput mapping method for RNA–RNA interactions. Extraction 2: A method for extracting terminal loop motifs from long and continuous RNAs. The extraction method comprehensively extracts single-terminal loop motifs defined as a single hairpin loop and its associated stem motifs (including internal loops and bulge loops). Subsequently, recognition of the combination of multiple single terminal loops can reconfigure and extract multi-terminal loops. The examples are representative motifs selected from the libraries conducted in this study (maximum length of the structure region: 116 nt). See also Supplementary Figs. 2–4.

FOREST is a system that identifies target-binding RNA structures with a quantitative score using an RNA structure library (Fig. 1). The datasets of FOREST enable an analysis of the binding properties of target molecules, comparisons of conditional interactions, and identifications of specific RNA structures. FOREST is composed of in silico and in vitro steps to generate the RNA structure library, multiplex affinity assay, and structure-wide evaluation by massively parallel detection on a microarray (Supplementary Fig. 1). We initially established an in silico pipeline to design the RNA library by extracting structured RNA motifs from large-scale datasets (Fig. 2). In this study, we focus on two types of RNA motifs as follows: (1) the validated stem–loop and (2) defined terminal motifs. As validated stem–loop motifs, we first extracted terminal loop motifs of human pre-miRNAs (hsa-mir) and a part of the miRNA region from miRBase (Fig. 2, Extraction 1, Supplementary Fig. 2)^12^. We chose these motifs because pre-miRNAs contain stem structures biologically validated by the recognition and processing by Dicer in cells and because the loop regions of some pre-miRNAs are known to regulate cell fate via RNP interactions^13–16^. As defined terminal motifs, we extracted single- and multi-terminal motifs consisting of one stem–loop motif with a blunt end without flanking sequences and consisting of two or more single-terminal motifs, respectively (Fig. 2, Extraction 2, Supplementary Fig. 3). The terminal motifs can serve as a unit that is compatible with the extraction of motifs from any RNA structure, including long non-coding (lnc)RNA and mRNA. The duplex groups in PARIS sequencing reads were used as a resource of validated stem structures in vivo (Supplementary Fig. 4)^9^.

**Fig. 3:**
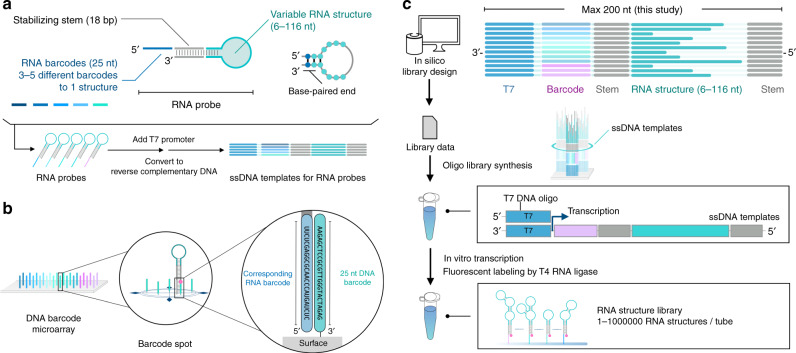
RNA structure library for ligand interactions and DNA barcode microarray for quantification. **a** In silico molecular design for the RNA structure library. Extracted RNA structures are conjugated to the stabilizing stem structure and RNA barcodes for massively parallel quantification of each RNA in the library. After the addition of the T7 promoter sequence, reverse complementary DNA sequences are synthesized as an oligo pool. See also Supplementary Fig. 1 and Methods. **b** Schematic of the barcode-based hybridization on a custom DNA microarray. Each spot on the microarray contains DNA sequences complementary to one barcode region of the RNA probe in the library. **c** In vitro preparation of the RNA structure library. The single-stranded DNA (ssDNA) templates in the pool have anti-T7 promoter sequences that can hybridize to the T7 promoter ssDNA. In turn, the T7 polymerase produces the RNA structures in a single tube. In vitro transcription generates the library in a single tube from the oligo DNA pool. The subsequent RNA ligation attaches a fluorescent dye to the 3′ end of the RNA probes for detection and quantification.

Next, to design RNA probes for the RNA structure library, we connected the extracted RNA structures with 25-mer RNA barcodes as molecular identifiers via a stabilizing stem structure (Fig. 3a). All extracted structures have a base-paired 5′–3′ end with few exceptions, hence, the attachment of the stabilizing stem structure extends the base-paired stem of the extracted motifs. The appropriately attached stem structure folds the desired structure and prevents interaction with the RNA barcode. We assigned three to five RNA barcodes to each RNA structure from the disclosed datasets of barcodes for the hybridization of nucleic acid^17^. After implementing in silico library design procedures, we synthesized the oligo pool of DNA templates with a T7 promoter sequence and complementary sequences of the RNA probes. The whole designed RNA structure library, which contained different RNA structures in a single tube, was transcribed in vitro from the oligo DNA pool, and the 3′-termini were fluorescently labeled (Fig. 3b, c).

For the quantification platform, we used a DNA barcode microarray whose spots had DNA sequences complementary to each RNA barcode in the library (Fig. 3b, c). This microarray provides a massively parallel and direct quantification of target-binding RNA structures without an RT or PCR step. Thus, by integrating the RNA structure library with multiplexed RNA–ligand interaction assays, we could evaluate the interaction networks on a large scale.

### Validation of FOREST

**Fig. 4:**
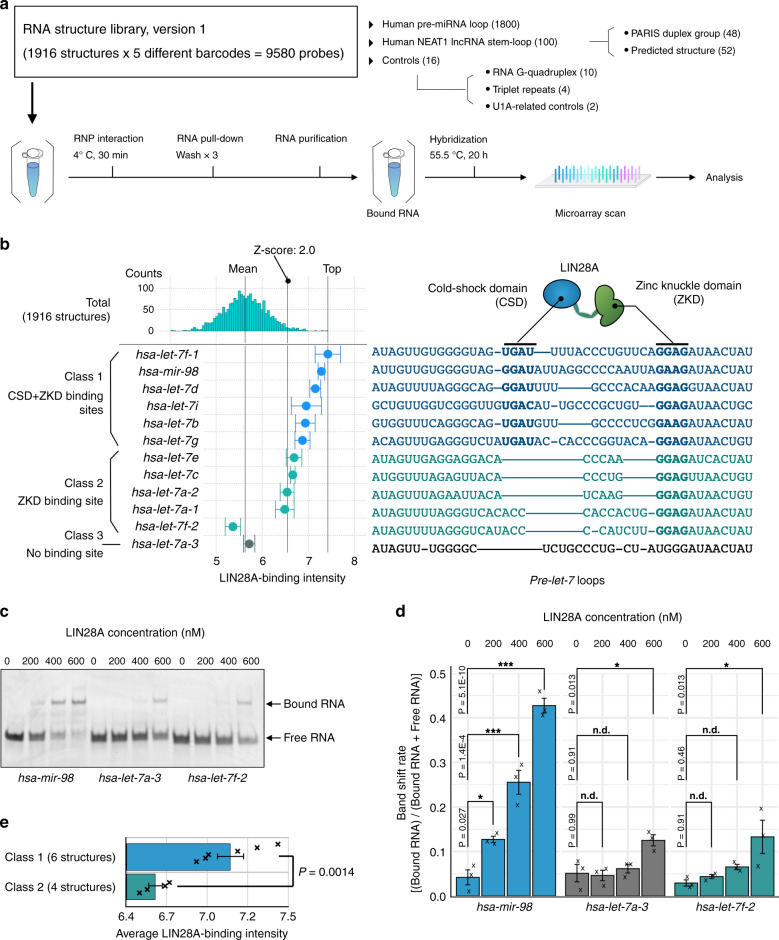
Massively parallel quantification of the RNP interactome using the RNA structure library. **a** Schematic of the RNA pull-down assay with the RNA structure library, version 1. The numbers of target RNA structures are described in parentheses. **b** FOREST analysis of *let-7* loops using RNA structure library, version 1 (1916 structures). The known *let-7* loops were categorized into three subclasses according to the presence of a cold-shock domain (CSD)-binding site and a zinc knuckle domain (ZKD)-binding site. The *X*-axis indicates the binding intensity of His-tagged LIN28A recombinant protein. The error bars indicate means ± s.d. of multiple barcodes assigned to each RNA. The additional lines indicate the top intensity, *Z*-score = 2.0, and mean. Binding intensities were calculated from the results of two independent experiments. The multiple alignments are taken from a previous study^21^. **c** Gel image of EMSA using 50 nM RNA. LIN28A solution was prepared by adjusting the concentration to 0, 200, 400, or 600 nM. The image shows representative data from three independent experiments. Source data are provided as a Source Data file. **d** The bar plots represent the band shift ratios observed in (**c**). The error bars indicate means ± s.e.m. The experiments were performed with three biological replicates. The *p*-values were determined by two-tailed Dunnett’s test. ***p < 0.001, **p < 0.01, *p < 0.05. n.d. indicates no significant difference. Each “×” indicates a data point. **e** Average LIN28A-binding intensities of *let-7* class-1 and class-2. We omitted the score of the *hsa-let-7f-2* loop from the calculation because it did not show a high intensity, unlike other class-2 loops. The numbers of analyzed *let-7* loops are described in parentheses. The error bars indicate means ± s.e.m., and the *p* values were determined by two-tailed *t* tests. Each “×” indicates a data point.

To validate FOREST, we performed the multiplexed detection of RNA structures and profiled RNP interactions between the RNA structure library and model RBPs. At first, we generated an RNA structure library, version 1 (v1), which includes 1800 human pre-miRNA loops, 100 structures of lncRNA (NEAT1), the validated U1A-related aptamer controls, rG4 controls, and triplet repeats (Fig. 4a). We confirmed the specificity and reproducibility of the barcode-based hybridization to the target and control spots on the DNA barcode microarray (Supplementary Fig. 5a, b). The fluorescence intensity from each barcode spot showed that the barcode-based hybridization possesses high orthogonality and specificity among a wide range of RNA amounts, except for the sample with the lowest amount, compared with control barcode spots (Supplementary Fig. 5c). We confirmed the reproducibility of the barcode-based hybridization based on the high correlation coefficients between two independent trials (Supplementary Fig. 5d, e). We also confirmed that the fluorescence intensities from the microarray spots were unbiased among all RNA classes, including triplet repeats and rG4 controls, indicating that the RNA structure library is transcribed consistently irrespective of the structural context (Supplementary Fig. 6).

To evaluate the affinities of RNP interactions, we performed RNA pull-down assays, quantified the amounts of enriched RNA probes with fluorescence, and calculated enrichment scores by subtracting the amounts of a control sample as the background noise and represented the scores as binding intensities (Figs. 1 and 4a). We first used the human U1A (SNRPA) protein and RNA structure library, v1. The known U1A-binding aptamer was significantly enriched and had the highest binding intensity, whereas its defective mutant did not show a significant score (Supplementary Fig. 7a, b). Sequence motif frequency analysis confirmed that the known U1A binding sequence^18,19^, 5′-GCAC-3′, was a vital factor for interacting with U1A (Supplementary Fig. 7c). Further analysis of the structural context revealed that U1A preferred the GCAC sequence on the loop region rather than the GCAC sequence on the stem region (Supplementary Fig. 7d, e). This result can explain why some GCAC-containing RNA structures were detected with low intensities. We validated the results with an electrophoretic mobility shift assay (EMSA) and confirmed that top-ranked RNA structures bound to U1A and that their U1A-binding intensities on FOREST correlated with their affinities (Supplementary Fig. 8). These results demonstrated that FOREST can quantitatively assess RNP interactions using thousands of RNA structures.

Next, we used FOREST to analyze the binding properties of LIN28A protein with the pre-miRNA loops. We focused on human *lethal-7* (*let-7*) pre-miRNA loops because previous studies have characterized them as LIN28-binding structures, except for *hsa-let-7a-3*^20^. We confirmed that ten out of twelve *let-7* loops interacted with LIN28A with significantly high affinities (Fig. 4b). FOREST and EMSA validation found that the *hsa-let-7f-2* loop did not interact efficiently with LIN28A under our conditions to a similar extent as the *hsa-let-7a-3* loop (Fig. 4c, d). Notably, we observed a significant difference in binding intensities depending on the presence of the cold shock domain (CSD)-binding sequences (Fig. 4e). These results coincided with a previous study that showed interaction with the CSD domain of LIN28A is a vital component for high-affinity binding to specific *let-7* loops and the downregulation of miRNA biogenesis in cells^21^. Collecting these results of human RBPs, we concluded that FOREST can generate the RNA structure library with proper folding and identify biologically functional RNA elements through binding intensities.

### Analysis of rG4 structure and its binding proteins

**Fig. 5:**
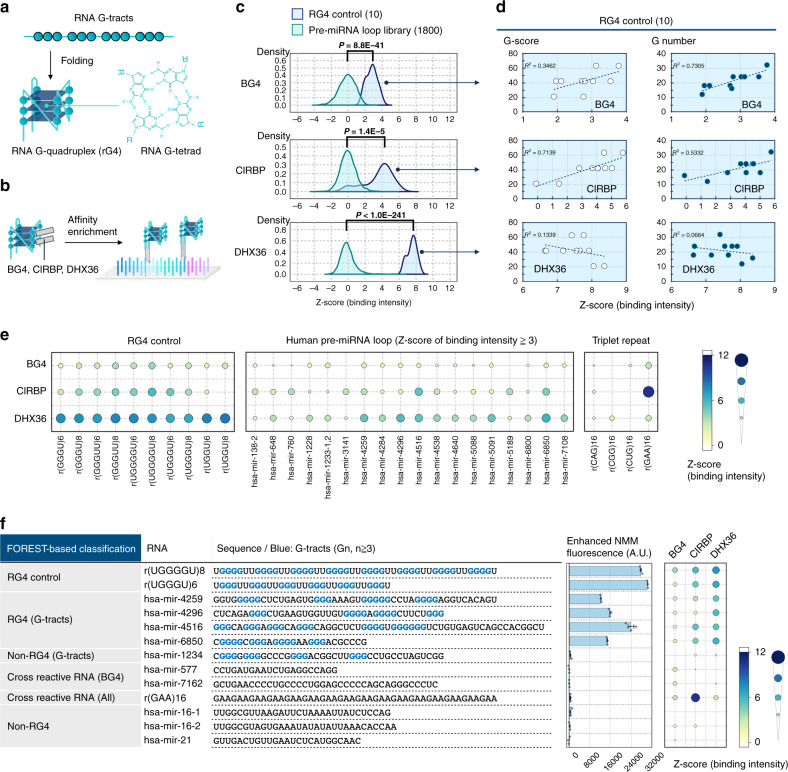
The binding properties of RNA G-quadruplex-binding proteins revealed by FOREST. **a** Schematic of an rG4 structure. A canonical rG4 is formed by four repeats of sequential guanine nucleotides. A monolayer of rG4 is called a “G-tetrad” and consists of Hoogsteen base pairing. **b** Schematic of the FOREST-based characterization of rG4-binding proteins (BG4, CIRBP, and DHX36). **c** Average BG4-binding intensities comparing rG4 controls and human pre-miRNAs. The error bars indicate means ± s.d.; the *p* value was determined by the two-tailed Brunner–Munzel test. The data were calculated from the average of two independent experiments. **d** Scatter plots show correlations between the number of guanine nucleotides or G-scores for the binding intensity of rG4 controls (ten species). **e** The binding intensities of representative RNA structures; rG4 controls, triplet repeats, and pre-miRNA loops with *Z*-scores ≧ 3 for at least one protein. The size and color of a dot represent the *Z*-score of the binding intensity. **f** In vitro rG4-sensing assays validated rG4. Blue characters highlight rG4-forming sequences. rG4 formation was analyzed by using N-methyl mesoporphyrin IX (NMM), which senses rG4 by enhanced fluorescence (*λ*_ex_ = 399 and *λ*_em_ = 610 nm). The error bars indicate means ± s.e.m.; the rG4-sensing assays were performed with three biological replicates, each with two technical replicates. Each “◇” indicates a data point. Source data are provided as a Source Data file.

To investigate whether we could analyze the interaction landscape between highly structured RNA and its binding proteins, we applied FOREST to identify rG4 in the library and the binding preferences of rG4-binding proteins. RG4 is a stable RNA structure composed of Hoogsteen base pairs (Fig. 5a), and it regulates gene expression and cell functions^22,23^. Several RBPs have been reported to interact with rG4^24–28^, but their binding preferences to rG4 and other RNA structures have not been fully explored. Sequencing-based methods have analyzed the rG4 structures and footprints of rG4-binding proteins^29–32^. However, it is not easy to compare the properties of rG4 candidates, because these methods cannot determine discrete motif boundaries. Quantitative comparisons of rG4-containing regions are also limited due to the RT and PCR amplification bias caused by structured or repetitive nucleic acids. We expected that FOREST would be suitable for the quantitative evaluation of rG4 and its binding proteins, because of the amplification-free and comprehensive analysis platform of the preset RNA structure library. Therefore, in combination with affinity-based rG4 structure enrichment, we elucidated the binding properties of three rG4-binding proteins as models: a Fab fragment of BG4 DNA/RNA anti-G4 antibody (BG4), cold-inducible RNA binding protein (CIRBP), and DEAH-box helicase 36 (DHX36)^24,26,28^. Fluorescence scanning on the barcode microarray directly quantified the bound RNAs without an RT or PCR step (Fig. 5b).

As a result, the average binding intensity of rG4 controls to the proteins was significantly higher than that of the pre-miRNA loop sublibrary (Fig. 5c), confirming that the proteins specifically bind to rG4. Among them, the specificity of the RNP interaction to the rG4 controls was highest for DHX36 and lowest for BG4 (Fig. 5c). This result indicated that the binding specificity differs among rG4-binding proteins. A comparison of binding scores found the relationship with the G-score, a measure of G4-forming sequences, and the G number (number of G nucleotides in the sequence) also revealed differences among the three proteins (Fig. 5d, Supplementary Note 1)^33^.

We also identified cross-reactive RNAs that do not have rG4-forming sequences but have high binding intensities to BG4, such as r(GAA)16 (Supplementary Figs. 9 and 10). Enriched sequence motif analysis also showed the presence of cross-reactions to BG4, because the sequence motif logo of BG4 is different from consecutive G-tracts like that of DHX36 and CIRBP (Supplementary Fig. 11, Supplementary Note 2). These results suggested that FOREST uncovered differences in specificity and preference among the rG4-binding proteins. In addition, FOREST distinguished cross-reactive RNAs and rG4 by a comprehensive comparison of the two datasets under conditions with different cations (Li^+^ vs. K^+^), which affect the stabilization of rG4 (Supplementary Fig. 12, Supplementary Note 3). This finding indicates that specific RNA structural changes induced by different buffer conditions caused differences in the BG4-binding intensities between rG4-forming and cross-reactive RNAs (Supplementary Fig. 12b, c).

Interestingly, we found that some pre-miRNA loops have high binding intensities, similar to those of the rG4 controls (Fig. 5e). For validation, we focused on two pre-miRNA loops containing potential rG4-forming G-rich sequences with high G-scores, because a simple comparison of the sequences could not distinguish the different binding intensities: *hsa-mir-6850* loop, which is bound to the three rG4-binding proteins (G-score = 42), and *hsa-mir-1234* loop, which also contained a G-rich sequence and high G-score (G-score = 37), but did not show high binding intensities to any of the proteins tested. We analyzed these two pre-miRNA loops using circular dichroism (CD) spectrum analysis and found that the *hsa-mir-6850* loop showed a typical CD pattern for rG4 that is dependent on the buffer conditions, whereas the *hsa-mir-1234* loop did not (Supplementary Fig. 13). The further analyses confirmed that the competition between Watson–Crick and Hoogsteen base-pairs could explain these binding differences, as shown in a previous report (Supplementary Figs. 14 and 15, Supplementary Note 4)^29^.

Next, we performed a fluorescence assay using N-methyl mesoporphyrin IX (NMM), which specifically detects G4 based on the fluorescence intensity^34,35^. The enriched RNAs that contain the canonical rG4-forming sequence (the pre-miRNA loops of *hsa-mir-4259*, *hsa-mir-4296*, *hsa-mir-4516*, and *hsa-mir-6850*) showed higher intensities than the non-rG4 controls, indicating that these pre-miRNA loops form rG4 structures (Fig. 5f). In contrast, the unenriched *hsa-mir-1234* loop and non-G4 structures (*hsa-mir-577* loop, *hsa-mir-7162* loop, and r(GAA)16) that cross-reactively bound to some rG4-binding proteins did not show enhanced NMM fluorescence intensity (Fig. 5f). Together, FOREST allowed quantitative analysis and discovery of rG4 structures from the library, elucidating the binding landscape of rG4-binding proteins.

### RNA structure-wide landscape of functional interactions

**Fig. 6:**
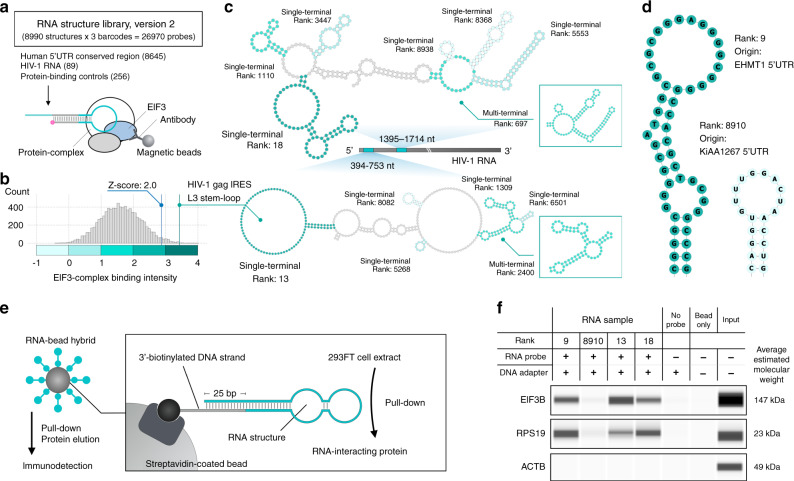
Systematic identification of protein-binding RNA elements from large-scale RNA structural architecture. **a** Schematic of RNA immunoprecipitation with an anti-EIF3B antibody and RNA structure library, version 2. The numbers of target RNA structures are described in parentheses. The 293FT cell-derived EIF3 complexes were immobilized on the antibody-conjugated beads. **b** RNP interactions of the EIF3 complex. The data were calculated from two independent experiments. A histogram of binding intensities with the positive control (green line) and *Z*-score = 2.0 (blue line) is shown. The terminal motif derived from HIV-1 gag IRES was a positive control. The colored bar indicates the binding intensities for the functional annotation. **c** A structure-wide functional annotation of the HIV-1 RNA secondary structures. The color of each structure was determined by the binding intensity; the library does not include RNA structures that contain gray-colored RNA residues. **d** Representative EIF3-complex binding structures from human 5′UTRs. **e** Schematic of the pull-down assay with an RNA–DNA–biotin hybrid. The target RNA structure was immobilized on a streptavidin-coated magnetic bead using a biotinylated tethering DNA strand. Enriched proteins in the bound fraction were collected by magnetic separation and analyzed by capillary-based immunodetection. **f** Gel images of the microcapillary-based immunodetection with anti-EIF3B, anti-RPS19, and anti-ACTB antibodies. The image shows representative data from two independent experiments. Average molecular weights were calculated from size markers and ladders. Source data are provided as a Source Data file.

To show the generality of FOREST, we expanded the library scale and demonstrated the identification of functional RNP interactions from long and continuous RNAs such as viral RNA and human mRNA. We designed RNA structure library, version 2 (v2), which contains the terminal motifs from the predicted secondary structure of human conserved 5’UTR regions and SHAPE-MaP datasets of the HIV-1 RNA genome (Fig. 6 and Supplementary Fig. 16)^5,36^. Using this library, we profiled the RNP interactions of the eukaryotic initiation factor 3 (EIF3) complex, which includes multiple proteins and regulates translation initiation in human cells (Fig. 6a, b). We successfully identified several binding elements from both the 5′UTR and HIV-1 RNA structures in the library (Fig. 6c, d). In the HIV-1 gag IRES, the known EIF3:40S-binding element (L3, Rank-13) was detected with a significant binding intensity^37,38^. Notably, we detected a high-affinity RNA element (Rank-18) from another region (1395–1714 nt) corresponding to upstream of the HIV-1 frameshift region. We validated that the high-ranked structures specifically pulled down EIF3B and an accompanying ribosomal protein from the cell extracts (Fig. 6f), demonstrating that the RNA structure library is useful for identifying functional interactions throughout continuous and long RNA.

**Fig. 7:**
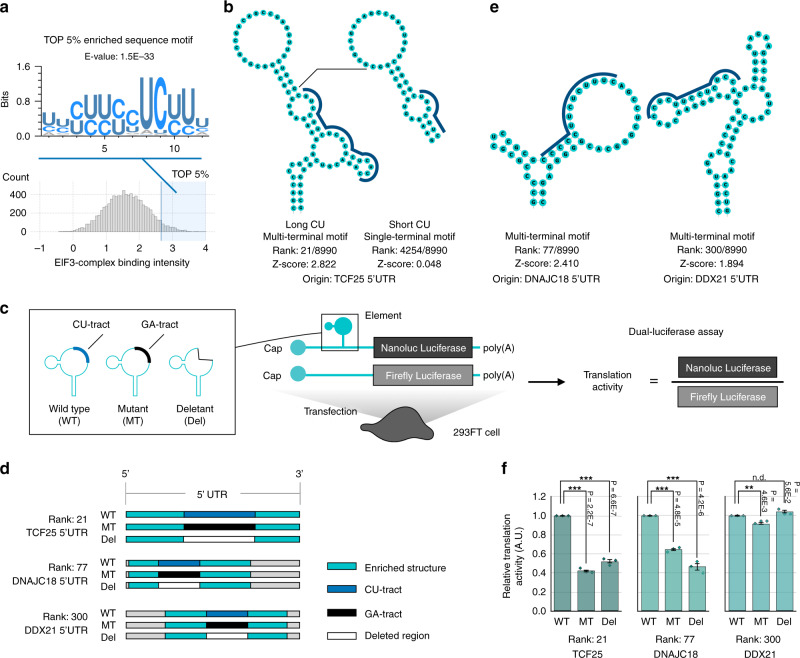
FOREST identifies translational regulatory elements by profiling the binding properties of the EIF3 complex. **a** An enriched sequence motif determined by MEME analysis of the population with the top 5% of cellular EIF3-complex binding intensities. **b** Two RNA structures derived from the 5′UTR of TCF25. **c** A dual-luciferase reporter assay using the designed 5′UTR of the identified candidates. A reporter mRNA encoding Nanoluc Luciferase was transfected into 293FT cells with Firefly Luciferase mRNA as the internal control. The CU-tracts of the RNA structures were redesigned as follows: mutants (MT) were designed by converting CU to GA sequences, and deletants (Del) were designed by deleting CU sequences. **d** Schematic of each 5′UTR of the reporter mRNAs. Reporter mRNAs contained the analyzed RNA structures with their whole 5′UTR sequences. The analyzed structures, the CU-tracts, and the converted GA-tracts are represented as turquoise, blue, and black regions, respectively. **e** Representative CU-rich RNA structures. According to the *Z*-scores, the DNAJC18-derived structure and the DDX21-derived structure have a high and medium affinity for the EIF3 complex, respectively. **f** Translational activity analysis. The intensities were normalized to those of each wild type. Error bars indicate means ± s.e.m. The experiments were performed with three biological replicates, each with two technical replicates. The *p* values were determined by two-tailed Dunnett’s test. ***p < 0.001, **p < 0.01, *p < 0.05. n.d. indicates no significant difference. Each “◇” indicates a data point. Source data are provided as a Source Data file.

Next we hypothesized that specific elements are involved in RNP interactions. In this context, sequence motif analysis confirmed that CU-tracts were significantly enriched in the top 5% of the population (Fig. 7a). Given the two elements derived from the same 5′UTR of *TCF25*, the longer CU-tract causes a higher intensity than the shorter one (Fig. 7b). To validate their functions, we next analyzed the translational effect of the 5′UTR elements using a dual-luciferase assay (Fig. 7c). We used Rank-21, Rank-77, and Rank-300 structures derived from the 5′UTRs of *TCF25*, *DNAJC18*, and *DDX21*, respectively, as representative elements (Fig. 7d, e). According to the *Z*-scores, we considered Rank-21 and Rank-77 as high-affinity elements and Rank-300 as medium-affinity elements. Interestingly, the CU-tract of Rank-21 and Rank-77 significantly facilitated cellular translation compared with their mutants, which delete or substitute the CU-tracts (Fig. 7f). In contrast, the CU-tract of the Rank-300 element did not strongly affect the translation. Collectively, FOREST revealed CU-tracts on the identified high-affinity elements to be critical factors for translation regulation in cells.

## Discussion

FOREST provides a high-throughput method for the quantitative assessment of transcriptome-wide structures using a synthetic library of genomically annotated, in silico designed, and validated or defined RNA structures (Fig. 1). The RNA structure library is scalable and customizable in terms of target RNA regions and motifs. Thus, FOREST can assess the function of the structures in a massively parallel manner by a multiplexed affinity assay.

The RNA structure library can characterize RNA-ligand interactions through a broad range of RNA classes and structured motifs. Compared with the randomized sequence libraries used to identify RNA motifs^18,39^, our RNA structure library provides feasible solutions for the requirements of structural variations and lengths and guarantees that the coded RNA structures exist in natural genomes. In contrast to the binding regions revealed by random fragment genomic libraries^40^ and CLIP-seq-based methods, FOREST uses RNA structures with discrete boundaries, enabling direct identification of the functional RNA structures from long and continuous transcripts without further examination (Figs. 6 and 7).

In the RNA structure library, each structure was tagged with an RNA barcode that can hybridize with its complementary barcode strand on a microarray for parallel quantification (Supplementary Fig. 5). The barcode-based hybridization of enriched RNA probes has advantages in the usage of target molecules free of fluorescent labels compared with current arrayed RNA technologies^41,42^. This feature realized the quantitative analysis of various types of ligands including protein complexes derived from cells (Fig. 6, Supplementary Fig. 17). In addition, because the barcode microarray directly detects the enriched RNA probes without structure-specific RT and PCR biases, FOREST can be used for massive analysis towards highly structured RNA such as rG4 (Fig. 5).

The vital aspect of the library design is the accuracy of the RNA datasets. It is known that the prediction of secondary structures only with a long RNA sequence does not always provide correctly folded structures^43^. Hence, to design the human 5′UTR sublibrary, we omitted longer RNA sequences (>400 nt) at the first step (Supplementary Fig. 16). High-throughput chemical structure probing methods improve the accuracy because prediction software can incorporate a large scale experimental dataset as the folding constraints. For example, SHAPE-MaP identified known RNA elements across the HIV-1 ssRNA genome, which were verified by other functional assays^44^. Other high-throughput methods, coupled with approximate ligation and/or cross-linking to fix RNA-RNA interactions, are also suitable for obtaining the validated RNA structures^7–10^. These methods can reproduce the features of 3D structures such as kissing loop, long-range duplex, and long-range interactions^9,10^. These results show that high-throughput structure probing is a reasonable approach to identify the RNA structure from long and continuous RNAs with sufficient accuracy.

In the affinity profiling step of FOREST, multiple reaction conditions can be tested to compare the biophysical and chemical effects on the function and structure of different RNA probes (Fig. 1, bottom right). We believe that the comparison of multiple conditions is one of the advantages of FOREST because other high-throughput methods (e.g., CLIP-seq) cannot easily change the conditions. For example, we examined the relationship between BG4 binding and conformational changes under various conditions, including different cations (Li^+^ vs. K^+^) (Supplementary Fig. 12) and a crowded environment with small molecules (Supplementary Fig. 15, Supplementary Note 4). Of note, we confirmed that the molecular crowding effect enhanced the BG4 binding intensity of specific RNA structures containing G-tract or binding sequences in base pairs (e.g., *hsa-mir-1234* loop). This observation suggested that physiological effects could dynamically alter the RNA structures in the library.

It is noteworthy that FOREST quantitatively evaluates noncanonical RNA structures across the RNA structure library. Because the current transcriptome-wide RNA structures were modeled with Watson-Crick base pairing, the landscape of noncanonical RNA structures is not well investigated. We applied FOREST to identify rG4 with discrete RNA regions using the RNA structure library, because there is no limitation regarding endogenous RNA resources, no amplification bias toward highly structured RNA with repetitive sequences, and no requirement for RNA random fragmentation or counting RT-stops to assess the formation of rG4. In fact, we succeeded in finding rG4-forming RNAs from the human pre-miRNA loops and different binding preferences among the tested rG4-binding proteins (Fig. 5). In addition, the direct identification of rG4 with a discrete boundary and quantitative score may provide a useful dataset for prediction software and further research, since the sequencing-based footprinting of rG4 cannot easily acquire this structural dataset^29,30^.

Our RNA structure library can be further improved by increasing the maximum RNA length and decreasing the intermolecular interactions between RNA probes. At the beginning of this study, the length limitation of oligo pools (Agilent Technologies) was 230 nt, but eventually extended to 350 nt (oPools, IDT). Given that the total length of the attachments is 84 nt, the current limitation of the RNA structure length is 266 nt in this condition. Considering the known binding sites, we assume that this maximum length is likely long enough to interact with most ligands. For example, 99.3% of the RNA structures of riboswitches (59404 out of 60290 RNAs) are less than 266 nt, according to RNAcentral, v15^45^. Even in longer RNAs, the terminal motif extraction method (Fig. 2) automatically divides the RNA structure into subordinary RNA motifs less than 266 nt. In addition to improving oligo library synthesis technology, multiplex oligo assembly such as DropSynth technology^46^ may solve the problem of synthesizing more extended DNA templates for the RNA structure library. As for decreasing the intermolecular interactions between RNA probes, we cannot rule out the possibility of intermolecular RNA-RNA interactions within the library. For example, multi-molecules of G4-forming sequences may form an rG4 structure by intermolecular RNA–RNA interactions. However, in this study, we did not observe a prominent multimer band for G4-forming RNA probes (e.g., *hsa-mir-6850*, r(UGGGU)6) in the gel images under native PAGE conditions (Supplementary Fig. 9, lanes for BG4 0 nM) or a difference in the fluorescent intensities of the rG4 controls compared with other RNA probes (e.g., the pre-miRNA loops) (Supplementary Fig. 6). These data suggested that the multimer states of the RNA probes including rG4 structures were not dominant in the tested library.

In conclusion, we developed FOREST and applied it to the comprehensive analysis of RNP interactions using diverse RNA structures extracted from multiple databases. In the future, FOREST will explore other functional RNA classes in addition to RNP interactions (Supplementary Discussion). These advances will lead to the elucidation of a regulatory layer that is governed by functional RNA elements.

## Methods

### In silico extraction of single-terminal and multi-terminal motifs

The inputs are RNA sequences and the secondary structures represented in dot-bracket format, and the output is the group of RNA motifs extracted from the RNA datasets. The following are procedures for the recognition of terminal motifs (Supplementary Fig. 3). (Step 1): All hairpin loop structures are extracted from the datasets of the RNA secondary structure. (Step 2): The stem structure linked to each hairpin loop structure is extracted by recognizing the base pair and the structural boundary, and the combination of the two is defined as a single-terminal motif and selected. During this procedure, the stem structure can include one or more bulge and/or loop structures. (Step 3): A multi-terminal motif is defined as a multi-branched stem–loop structure containing more than two single-terminal motif structures. (Step 4): If a target RNA motif is longer than the RNA length limitation, the motif will repeatedly shorten to the maximum length or the nearest base-paired end. The selected terminal motif is adopted as an output when the length of the selected RNA is less than the synthetic length limit.

### Design of RNA structure library, version 1: validated stem–loop library

Pre-miRNA loop sublibrary: Human pre-miRNA sequences and their secondary structures were obtained from miRBase version 21 (Supplementary Fig. 2)^12^. We defined a pre-miRNA loop region as follows. (1) Start and end nucleotides should be in the same column on the secondary structure of the pre-miRNA presented in miRBase. (2) If the pre-miRNA produces one mature miRNA, the start nucleotide of the loop region is set as the fourth nucleotide from the loop-side end of the mature miRNA sequence. (3) If the pre-miRNA produces two mature miRNAs, then the strand closer to the basal end is chosen, and the loop region is set as in (2). If the same loop motifs are confirmed from more than two pre-miRNA genes, the pre-miRNA loop is enrolled in the library with annotation to all original pre-miRNA genes. Following these parameters, we obtained 1805 human pre-miRNA loops. Because of the length limitation (86 nt), we omitted the five pre-miRNA loops from the library (hsa-mir-3648-1_3648-2, hsa-mir-3652, hsa-mir-3976, hsa-mir-6753, and hsa-mir-6892).

NEAT1 lncRNA sublibrary from PARIS datasets: To design the RNA structure library from NEAT1 lncRNA, we extracted sequence reads from the PARIS data of HeLa cells^9^. The in silico pipeline previously described^9^ was used to generate duplex groups. The reads of the duplex group were mapped to hg38 reference genome. The gapped reads of the duplex group were used for the following processes. Due to the capacity of the library scale, we focused on duplex groups that mapped to previously identified functional regions (9.7–11, 12–13, and 15.4–16.6 k) of lncRNA NEAT1^47^. The structures were obtained by RNAcofold^48^ with default parameters with the input of both the right and left arms of the PARIS duplex groups. Extracted motifs were filtered according to the length (nt). In the case of the RNA structure library, v1, the length limitation was set to 86 nt. The terminal motifs were extracted by removing the flanking regions. Following these procedures, we obtained 48 motifs for RNA structure library, v1.

NEAT1 lncRNA sublibrary from ParasoR datasets: ParasoR software was used to predict the secondary structure of the full-length RNA and functional regions^49^. The inputs of ParasoR were the full length of NEAT1 and the three functional regions (9.7–11, 12–13, and 15.4–16.6 k). The terminal-loop motifs were extracted from the predicted structures. Extracted motifs were filtered according to the length (nt). In the case of the RNA structure library, v1, the length limitation was set to 86 nt. In the case of full-length RNA, we removed the extracted RNA motifs that were not localized at the functional regions. Following these procedures, we obtained 52 motifs for RNA structure library, v1.

### Design of RNA structure library, version 2: terminal stem–loop library

Human 5′UTR sublibrary: To design the RNA structure library from the human 5′UTR, we extracted RNA sequences from the human 5′UTR registered in UTRdb^36^. To obtain structural information, the RNA secondary structures of the full-length 5′UTR sequences were predicted by CentroidFold v.0.0.15^50^. To maintain the precision of the prediction, we removed 5′UTR sequences longer than 401 nt from the library. The option gamma was set to 4. The terminal motifs were extracted as mentioned above. To focus on sequences conserved among species, RNA terminal motifs with conserved sequences were preferentially included in the RNA structure library. We used the annotations of the conserved regions registered in UTRdb. Due to the scale of the library synthesis, the library was adjusted to contain 8645 types of terminal motifs. These motifs were loaded into RNA structure library, v2.

HIV-1 structure sublibrary: To design the RNA structure library from HIV-1 RNA, we obtained RNA structure datasets determined by SHAPE-MaP with structural analysis^5^. The datasets were converted to RNA secondary structure information without pseudoknots using RemovePseudoKnots software on the RNA structure web server^51,52^. In addition, using ct2dot software on the RNA structure website, the secondary structure information was obtained in a dot-bracket format. Then, the structures were divided into terminal motifs, and all motifs were loaded into RNA structure library, v2.

Protein-binding controls: As controls, selected pre-miRNA loops, protein-binding RNA aptamers, and defective mutants were loaded into RNA structure library, v2.

### Oligo template pool and DNA barcode microarray design

To generate the RNA structure library, single-stranded DNA sequences were used as templates for RNA probes, including each RNA structure extracted from the datasets. The templates were designed to include five components in the following order from the 3′ end.

- CC + T7 promoter sequence: A sequence for synthesizing RNA from template DNA. The sequences of the following components were transcribed using T7 polymerase.
- Barcode sequence (25 nt): To immobilize each RNA probe on the corresponding microarray spots, the barcode sequences were orthogonally hybridized with the complementary strands of the barcode sequences placed on the DNA microarray. The sequence information of these orthogonal barcodes is listed elsewhere^17^. In the RNA structure library, multiple barcode sequences were assigned to each RNA structure.
- Stabilizing stem structure sequence Forward (18 nt): A unique sequence that forms a stem structure to stabilize the RNA structure and insulate the RNA structure and RNA barcode sequence.
- RNA structure region (6-85 nt: v1, 6-116 nt: v2): Variable RNA motifs extracted from RNA structure datasets.
- Stabilizing stem structure sequence Reverse (18 nt): A stem structure constructed by base-pairing with the stabilizing stem structure sequence Forward.

These template DNAs were synthesized by OLIGONUCLEOTIDE LIBRARY SYNTHESIS (OLS, Agilent Technologies). The size of the oligo templates was limited to 170 and 200 nt for RNA structure libraries v1 and v2, respectively. After the assignment of the barcodes to the RNA structure, we converted the barcodes to antisense DNA strands. The listed DNA strands were submitted to the SureDesign server as CGH custom array design services (Agilent Technologies). Probe Replication Factor was set to 5× and 2× for RNA structure library, v1, and v2, respectively. Then, a custom CGH DNA microarray was purchased in the 8× 60 K array format.

### In vitro transcription of RNA probes and the RNA structure libraries

To produce short RNA probes (less than 300 nt) and the RNA structure libraries, we used the MEGAshortscript T7 Transcription Kit (Thermo Fisher Scientific) following the manufacturer’s instructions. Twenty microliters of the reaction solution containing single-stranded DNA templates and single-stranded DNA coding T7 promoter sequence (5′-GCGCTAATACGACTCACTATAGGG-3′ for RNA structure library, v1 or 5′-CCGCGCTAATACGACTCACTATAG-3′ for RNA structure library, v2) was prepared and incubated at 37 °C. The reaction time was set to 20 h for the transcription of the RNA structure libraries and 6 h for the others. Then, 2 μL of TURBO DNase (Thermo Fisher Scientific) was added to the reaction solution, mixed by pipetting, and incubated at 37 °C for 15 min. RNA products were purified with Zymo RNA Clean and Concentrator (Zymo Research).

### 3′-Terminal Cy5 labeling

For detection and quantification on the microarray, all RNA probes in the library were labeled with fluorescent dye at the 3′ end by mixing 1× T4 Ligase Buffer (Thermo Fisher Scientific), 100 μM pCp-Cy5 (Jena Bioscience), 10 μM RNA structure library, and 0.5 U/μL T4 RNA Ligase (Thermo Fisher Scientific) in a 100 μL reaction mixture. The mixture was incubated at 16 °C for 48 h under light-shielded conditions. The labeled RNA probes in the library were purified with Zymo RNA Clean and Concentrator (Zymo Research). The fluorescently labeled RNA structure library was stored at −28 °C.

### Hybridization and microarray scanning

Eighteen microliters of enriched RNA was mixed with 4.5 μL of 10× Blocking Agent (Agilent Technologies) and 22.5 μL of Hi-RPM Hybridization Buffer (Agilent Technologies). The samples were incubated for 5 min in a heat block set at 104 °C. Then, the samples were immersed in ice water and incubated for 5 min. The samples were applied to an 8× 60 K Agilent microarray gasket slide (Agilent Technologies). The gasket slide and CGH custom array 8× 60 K (Agilent Technologies) were assembled with SureHyb. In a hybridization oven (Robbins Scientific), hybridization was performed for 20 h at a temperature of 55.5 °C at 20 rpm. After hybridization, the microarray slide was washed for 5 min with Gene Expression Wash Buffer 1 (Agilent Technologies) in a glass container at room temperature. Next, the microarray slide was transferred to a glass container containing Gene Expression Wash Buffer 2 (Agilent Technologies), which was immersed in a thermostat bath at 37 °C and washed for 5 min. Finally, we used SureScan (Agilent Technologies) to obtain fluorescence image data on the microarray. The captured images of the microarray slide were converted to numeric fluorescence intensities of each spot by Feature Extraction (Agilent Technologies) and GeneSpringGX (Agilent Technologies).

### RNA pull-down with His-tagged human proteins (U1A, LIN28A)

His-U1A protein was purified as detailed in our previous paper^53^. His-LIN28A protein was a gift from Dr. Kozo Tomita. His-tagged protein (20 pmol of LIN28A, 5 pmol of U1A protein), 20 μL of TALON magnetic beads (Clontech), and 1 μg of the RNA structure library were mixed in 1 mL of protein-binding buffer (20 mM HEPES pH 7.5, 80 mM KCl, 20 mM NaCl, 10% glycerol, 2 mM DTT, and 0.1 μg/μL BSA). To optimize the concentration, we first examined three protein amounts (5, 20, and 100 pmol). The optimum concentration was set as the minimum concentration, where the fluorescence intensity was within the dynamic range. A mixture containing no protein was also prepared as a control. The mixture was incubated on a rotator at 4 °C for 30 min and washed with 1× protein-binding buffer three times. Then, 200 μL of elution buffer (1% SDS, 10 mM Tris-HCl, 2 mM EDTA) was added to the magnetic beads, and the mixture was heated at 95 °C for 3 min. The RNA was collected from the supernatant by removing the magnetic beads. The RNA structure library in the mixture was extracted with phenol and chloroform with ethanol precipitation for purification.

### RNA pull-down with rG4 binding proteins (BG4, CIRBP, DHX36)

His-tagged BG4 anti-G4 antibody Fab fragment was purchased from Absolute Antibody (Ab00174-1.6). His-tagged CIRBP human recombinant protein was purchased from BioVision (7589-20). C-Myc/DDK-tagged (FLAG-tag compatible) DHX36 human recombinant protein was purchased from OriGene Technologies (TP323929). The RNA structure library was prepared in 1× K^+^ folding buffer (10 mM Tris-HCl pH 7.5, 100 mM KCl) or 1× Li^+^ folding buffer (10 mM Tris-HCl pH 7.5, 100 mM LiCl). For folding, RNA was heated at 95 °C and cooled to 4 °C at −6 °C/s on a ProFlex Thermal Cycler (Thermo Fisher Scientific). For this purpose, a target protein (100 pmol of BG4, 20 pmol of CIRBP, or 20 pmol of DHX36), 20 μL of TALON magnetic beads (Clontech) or 20 μL of the protein G dynabeads (Thermo Fisher Scientific, #DB10003) that were preincubated with 10 μg of monoclonal ANTI-FLAG^®^ M2 antibody (Sigma-Aldrich, #F1804) and 1 μg of the refolded RNA structure library were mixed in 1 mL of 1× BG4 protein-binding buffer (10 mM Tris-HCl pH 7.5, 100 mM KCl or 100 mM LiCl, 10% glycerol, and 0.1 μg/μL BSA). To optimize the concentration, we first examined three protein amounts (5 pmol, 20 pmol, and 100 pmol). The optimum concentration was set as the minimum concentration, where the fluorescence intensity was almost within the dynamic range. A mixture containing no protein was also prepared as a control. The mixture was incubated on a rotator at 4 °C for 30 min and washed three times with 1× BG4 protein-binding buffer. Then, 200 μL of elution buffer was added to the magnetic beads, and the mixture was heated at 95 °C for 3 min. The RNA was collected from the supernatant by removing the magnetic beads. The RNA structure library in the mixture was extracted with phenol and chloroform with ethanol precipitation for purification.

### Calculation of binding intensities

The protein-binding intensities of each RNA probe were calculated by subtracting the fluorescence intensities of the no-protein control samples from those of the enriched protein samples. To alleviate the effect of undesired interactions with the barcode region, we calculated the averaged fluorescence intensities of each RNA structure from the intensities of three RNA probes that had the same RNA structure but different RNA barcodes. In the case of RNA structure library, v1, because there were five barcodes per RNA structure, we excluded the maximum and minimum intensities from a set of five intensities.

### Electrophoretic mobility shift assay (EMSA)

U1A and LIN28A: First, 1000 nM RNA samples were prepared in 1× folding buffer (10 mM HEPES pH 7.5, 80 mM KCl, and 20 mM NaCl). For folding, RNA was incubated at 95 °C for 5 min and then immediately placed in tubes on ice. Then, 50 nM refolded RNA and recombinant proteins (U1A: 0, 20, 40, 80 nM, LIN28A: 0, 200, 400, 600 nM) were mixed in 10 μL of 1× protein-binding buffer. The mixtures were incubated at 4 °C for 30 min for U1A or 90 min for LIN28A. Then, 2.5 μL of 5× Hi-Density Sample Dye (Thermo Fisher Scientific) was added and mixed. The mixtures were overlaid on a nondenaturing 4-20% polyacrylamide gel (Thermo Fisher Scientific) at 4 °C. After electrophoresis at 10 mA for 50 min, the gel was stained with SYBR Green II. Imaging was performed with a Typhoon FLA7000 (GE Healthcare). Details of the RNA probes are available in Supplementary Table 1.

BG4 anti-G4 antibody Fab fragment: For this purpose, 500 nM RNA samples were prepared in 1× K^+^ folding buffer (10 mM Tris-HCl pH 7.5, 100 mM KCl). For folding, RNA was heated at 95 °C and cooled to 4 °C at −6 °C/s on a ProFlex Thermal Cycler. Refolded RNA was then incubated at 4 °C for at least 30 min. Then, 50 nM refolded RNA and 0 or 750 nM BG4 were mixed in 12 μL of 1× K^+^ BG4 protein-binding buffer (10 mM Tris-HCl pH 7.5, 100 mM KCl, 10% glycerol, 0.1 μg/μL BSA). Next, the mixtures were incubated at 4 °C for 90 min. Then, 3 μL of 5× Hi-Density Sample Dye (Thermo Fisher Scientific) was added and mixed. The mixtures were overlaid on a nondenaturing 6% polyacrylamide gel (Thermo Fisher Scientific) at 4 °C. After electrophoresis at 15 mA for 30 min, the gel was stained with SYBR Green I. Imaging was performed with a Typhoon FLA7000. Details of the RNA probes are available in Supplementary Table 2.

### Image analysis of EMSA

The band intensities were calculated from the gel images with three biological replicates. The gel images were analyzed using the Gels submenu in ImageJ (NIH). First, the lanes that each enclosed a shifted band and non-shifted band were defined, and the lane profile plots were generated. Then, peak areas were defined based on the bands of the corresponding gel images and measured as band intensities. For EMSA using LIN28A and U1A, the relative intensity of the shifted band compared with that of the non-shifted band in each lane was calculated. For EMSA using BG4, the relative intensity of the non-shifted band in the BG4 (+) lane compared with that in the BG4 (−) lane was calculated for each RNA probe. Relative intensities were normalized to non-shifted bands in the BG4 (−) lanes. In the gel image of *hsa-mir-98* loop, we excluded the weak intensity that did not show dose-dependent interaction and the intensity for the 2:1 complex of LIN28A:hsa-mir-98, since the intensities were quite low and difficult to distinguish from the background noise.

### RNA secondary structure visualization

We used the forna website [http://rna.tbi.univie.ac.at/forna/]^54^ to generate illustrations of the RNA secondary structures by modifying the colors with Adobe Illustrator.

### Calculation of G-scores

We used the QGRS website [http://bioinformatics.ramapo.edu/QGRS/help_search.php]^33^ to calculate G-scores and used the maximum G-score of the inputted RNA sequences for the analysis.

### Sequence motif discovery

We used the MEME website [http://meme-suite.org/tools/meme]^55^ and the Weblogo3 website [http://weblogo.threeplusone.com/create.cgi]^56^ to generate the sequence logo of enriched motifs from the top 5% of the population in the case of RNA structure library, v2. The k-mer (*k* = 4) counting was performed with the FOREST datasets of the top 5% (enriched RNA) and the whole population (all) to identify protein-binding sequence motifs from RNA structure library, v1 (Supplementary Fig. 11, Supplementary Note 2). The count of each k-mer was normalized by the sum of all counts. With *k* denoting each k-mer, the following formula defines relative frequency: relative frequency (*k*) = normalized Count (*k*)_Enriched_/normalized Count (*k*)_All_. Then, we collected the 4-mers whose *Z*-score for the relative frequency (*k*) was >2.58. Clustal Omega [https://www.ebi.ac.uk/Tools/msa/clustalo/]^57^ aligned the collected 4-mers with default parameters. The enriched sequence motif logo was generated from the aligned 4-mers by Weblogo3.

### N-Methyl mesoporphyrin IX (NMM) fluorescence assay

N-Methyl mesoporphyrin IX (NMM, Frontier Scientific) was purchased and resuspended in 1× K^+^ folding buffer. All RNAs were generated by in vitro transcription with a MEGAshortscript T7 Transcription Kit. RNA samples (1000 nM) were prepared in 1× K^+^ folding buffer. For folding, RNA was incubated at 95 °C and cooled to 4 °C at −6 °C/s on a ProFlex Thermal Cycler. Refolded RNA was then folded at 4 °C for at least 30 min. Then, 15 μL of refolded RNA (1000 nM) was mixed with 15 μL of NMM (100 μM) in 1× K^+^ folding buffer. The fluorescence was measured on Infinite (TECAN) with an excitation wavelength of 399 nm and an emission wavelength of 610 nm. The fluorescence intensity was corrected by subtracting the intensity of the buffer mixture as a background. Details of the RNA probes are available in Supplementary Table 2.

### Cell culture

293FT cells (Thermo Fisher Scientific) were cultured at 37 °C in 5% CO_2_ in DMEM with high glucose (Nacalai Tesque) containing 10% fetal bovine serum (Japan Bio Serum), 0.1 mM nonessential amino acids (Thermo Fisher Scientific), and 1 mM sodium pyruvate (Sigma-Aldrich).

### Protein collection by cell lysis

293FT cells were seeded in 10 cm dishes and cultured until 80–90% confluency. As a washing step, 10 mL of chilled phosphate-buffered saline (PBS) (4 °C) was added and removed immediately by an aspirator. This washing step was repeated again, and 5 mL of PBS was added. The cells were detached with a cell scraper, and the solution was transferred to a 50 mL tube. The supernatant was removed after centrifugation at 300 × *g* for 5 min at 4 °C. The cell pellet was resuspended in chilled PBS and centrifuged again at 300 × *g* for 5 min at 4 °C. The supernatant was carefully removed. Then, 900 μL or NP40 Cell Lysis Buffer (Thermo Fisher Scientific), 100 μL of 10× Protease Inhibitor Cocktail (Sigma-Aldrich), and 1 μmol of PMSF were added. Next, the mixture was incubated on ice for 30 min. During this time, the mixture was vortexed for 10 s every 10 min. Then, the mixture was centrifuged for 10 min at 13,000 rpm and 4 °C. The proteins in the supernatant were collected and stored at −80 °C in 1.5 mL Protein LoBind Tubes (Eppendorf) until use.

### RNA immunoprecipitation using antibodies and cell lysates

The Dynabeads Protein Immunoprecipitation Kit (Thermo Fisher Scientific) was used following the manufacturer’s instructions with modifications. Twenty microliters of Dynabeads was prepared in 200 μL of Ab Binding buffer (Thermo Fisher Scientific) with 10 μL of rabbit anti-EIF3B antibody (Bethyl Laboratories, A301-761A) or 10 μL of M2 mouse monoclonal antibodies to FLAG-tag (Sigma, F1804). As a control, we prepared a mixture that did not contain the target antibody. The mixture was incubated at room temperature for 13 min with a tube rotator. The tube was placed on a magnetic rack, and the supernatant was removed. Then, 200 μL of binding/washing buffer (Thermo Fisher Scientific) and 200 μL of the collected 293FT cell extracts (1 μg/μL) were added to the mixture. The mixture was incubated on a tube rotator for 1 h at 4 °C. After the reaction, the tube was placed on a magnetic rack, and the supernatant was removed. A washing step was performed with 1 mL of 1× protein-binding buffer. The tube was placed on a magnetic rack, and the supernatant was removed. Then, 500 μL of 1× protein-binding buffer and 500 ng of Cy5-labeled RNA structure library, v2, were added. The mixture was incubated on a tube rotator at 4 °C for 1 h. Another washing step was performed with 500 μL of the protein-binding buffer, and the supernatant was removed. Washing was repeated three times. Finally, protein-binding RNAs were recovered by phenol–chloroform extraction and ethanol precipitation.

### Pull-down assay

Twelve micromolar RNA probes were prepared in 1× hybridization buffer (20 mM HEPES-KOH pH 7.5, 80 mM KCl, and 20 mM NaCl) in the presence of 10 μM biotinylated DNA adapter. Then, RNA and DNA were incubated at 95 °C and cooled to 4 °C at −0.1 °C/s on a ProFlex Thermal Cycler to form RNA–DNA–biotin hybrids and mixed with 50 μL of Streptavidin Mag Sepharose (GE Healthcare) in 500 μL protein-binding buffer in 1.5 mL tubes. After incubation for 3 h at 4 °C with rotation, the RNA–DNA complexes on the beads were washed with protein-binding buffer. Then, 2 μL of RNase Inhibitor, Murine (New England Biolabs, M0314), 700 μL of 1× protein-binding buffer, and 200 μL of protein extract (1 μg/μL) derived from 293FT was added to the mixture. After incubation for 12 h at 4 °C with rotation, the protein–RNA–DNA complexes on the beads were washed three times with 1× protein-binding buffer. The RBP was detached by heating at 95 °C for 5 min with 5× Sample Buffer (ProteinSimple, 042-195). The enriched proteins were collected by removing the magnetic beads. Target proteins were detected by an automated capillary-based immunodetection system (Wes SimpleWestern, ProteinSimple) using 20-fold diluted anti-EIF3B (Bethyl Laboratories, A301-761A), 50-fold diluted anti-RPS19 (Bethyl Laboratories, A304-002A), and 50-fold diluted anti-ACTB (Cell Signaling Technology, 8457S) as primary antibodies. HRP-conjugated anti-rabbit (ProteinSimple, 042-206) was used as the secondary antibody. Details of the DNA adapter and the RNA probes are available in Supplementary Table 3.

### Preparation of capped mRNA

The 5′UTRs of reporter RNA were synthesized by GeneArt (Thermo Fisher Scientific). Templates were prepared by Fusion PCR using the 5′UTR variants, ORFs and common 3′UTR with a poly-A tail. Capped mRNAs were prepared by using the MegaScript Kit (Thermo Fisher Scientific) in the presence of an anti-reverse cap analog (TriLink BioTechnologies). Ten microliters of the reaction solution was prepared and incubated at 37 °C. After 6 h of incubation, 1 μL of TURBO DNase (Thermo Fisher Scientific) was added to the reaction solution, mixed by pipetting, and incubated at 37 °C for 30 min. RNA products were purified with Zymo RNA Clean and Concentrator (Zymo Research). Then, to remove the phosphate at the 5′ terminus, the RNA products were treated with Antarctic Phosphatase (New England Biolabs). The RNA products were purified again, and the concentrations were measured with a NanoDrop 2000 (Thermo Fisher Scientific). Sequence information regarding all DNA materials for constructing the templates of reporter mRNA is available in Supplementary Data 1.

### Dual-luciferase assay

293FT cells were prepared in 24-well plates and transfected with 50 ng of reporter mRNAs encoding Nanoluc Luciferase and 10 ng of inner control mRNA encoding Firefly Luciferase using Lipofectamine messengerMAX (Thermo Fisher Scientific) following the manufacturer’s instructions. To avoid saturation, Nanoluc Luciferase was added to the PEST-tag to promote protein degradation. After 13 h, the plates were placed on ice, and the cells were washed twice with chilled PBS. Then, 200 μL of passive lysis buffer (Promega) was added and mixed by pipetting. Supernatants were collected in 1.5 mL Protein LoBind Tubes and stored at −28 °C until measurement. We used the Nano-Glo Dual-Luciferase Reporter Assay System (Promega, N1610) following the manufacturer’s instructions to measure the luminescence. The luminescences were acquired on a GloMAX Navigator System (Promega). Translation activity was calculated as a ratio of the luminescence of Nanoluc Luciferase and of Firefly Luciferase (Fig. 7c). All samples were technically duplicated and biologically triplicated.

### Reporting summary

Further information on research design is available in the Nature Research Reporting Summary linked to this article.

## Supplementary information

Supplementary Information

Reporting Summary

Description of Additional Supplementary Files

Supplementary Data 1

Supplementary Data 2

Supplementary Data 3

Supplementary Data 4

Supplementary Data 5

Supplementary Data 6

Supplementary Data 7

Supplementary Data 8

Supplementary Data 9

Supplementary Data 10

Supplementary Data 11

Supplementary Data 12

Supplementary Data 13

## Data Availability

The datasets of FOREST are available in Supplementary Data 2–13. The source data underlying Figs. 4c, 5f, 6f, and 7f and Supplementary Figs. 8a, 9a, and 10b are provided as a Source Data file. To design RNA structure library version 1, we extracted pre-miRNA loops from miRBase ver.21^12^. Additionally, we extracted terminal motifs of NEAT1 from the predicted RNA secondary structures (Supplementary Data 13) and the PARIS dataset of HeLa cell^9^. To design RNA structure library version 2, we extracted terminal motifs from the UTRdb^36^ and SHAPE-MaP dataset of HIV-1 genome^5^. To design a barcode microarray, we used the datasets of barcodes for the hybridization of nucleic acid^17^. The data supporting the findings of this study are available from the corresponding authors upon reasonable request. Source data are provided with this paper.

## Source data

Source Data

## Code availability

The custom scripts for the motif extraction of the terminal motifs, designing the RNA structure library, and generation of the microarray ordering template are available in the Github page with the instruction to order as products (https://github.com/KRK13/FOREST2020/). The other codes used in this study are available from the corresponding authors upon request.
