# Supplementary material for: RNA structure-wide discovery of functional interactions with multiplexed RNA motif library
Source: Nat Commun. 2020 Dec 8;11:6275. doi: 10.1038/s41467-020-19699-5 (PMC7723054; doi:10.1038/s41467-020-19699-5)
Supplement: Supplementary file 1 — Supplementary Information [file 41467_2020_19699_MOESM1_ESM.pdf]

Supplementary Information

RNA structure-wide discovery of functional interactions with multiplexed RNA motif library

Kaoru R. Komatsu[1], Toshiki Taya[2], Sora Matsumoto[1], Emi Miyashita[1], Shunnichi Kashida*[1] and Hirohide Saito*[1]

[1]Department of Life Science Frontiers, Center for iPS Cell Research and Application, Kyoto University, 53 Kawahara-cho, Shogoin, Sakyo-ku, Kyoto 606-8507, Japan
[2]Twist Bioscience, 681 Gateway Blvd South, San Francisco, CA 94080, USA

*Correspondence:
shunnichi.kashida@gmail.com (S.K.) and hirohide.saito@cira.kyoto-u.ac.jp (H.S.)

Contents

Supplementary Figures

Supplementary Fig. 1 | Experimental workflow of FOREST
Supplementary Fig. 2 | Design of pre-miRNA loop library
Supplementary Fig. 3 | Definition of terminal motifs as target RNA structures
Supplementary Fig. 4 | Motif extraction from duplex map datasets
Supplementary Fig. 5 | Fluorescence signal scanning on DNA barcode microarray
Supplementary Fig. 6 | Confirmation of detection bias for each RNA class and RNA secondary structure
Supplementary Fig. 7 | Validation of FOREST using U1A-binding RNA structures
Supplementary Fig. 8 | Validation of U1A-binding RNA structures by EMSA
Supplementary Fig. 9 | EMSA using cross-reactive RNAs with a Fab fragment of BG4 DNA/RNA anti-G4 antibody
Supplementary Fig. 10 | Validation of BG4-binding RNA structures by ELISA
Supplementary Fig. 11 | Sequence motif analysis with FOREST datasets
Supplementary Fig. 12 | Evaluation of BG4-binding properties under different conditions in rG4 stabilization
Supplementary Fig. 13 | Validation of RNA structures by circular dichroism (CD) analysis of RNA
Supplementary Fig. 14 | Sequence-based classification of RNA G-quadruplex and relation to BG4-binding intensities
Supplementary Fig. 15 | Functional remodeling of RNA structures in a crowded environment with small molecules
Supplementary Fig. 16 | Design of human 5'UTR and HIV-1 library
Supplementary Fig. 17 | A rapid and general method using cell lysate and Flag-tags

Supplementary Tables

Supplementary Table 1. RNA probe sequences related to LIN28A or U1A
Supplementary Table 2. RNA probe sequences related to rG4-binding proteins
Supplementary Table 3. RNA probe sequences related to Fig. 6

Supplementary Notes

Supplementary Discussion

Supplementary Methods

Supplementary References

Supplementary Datasets

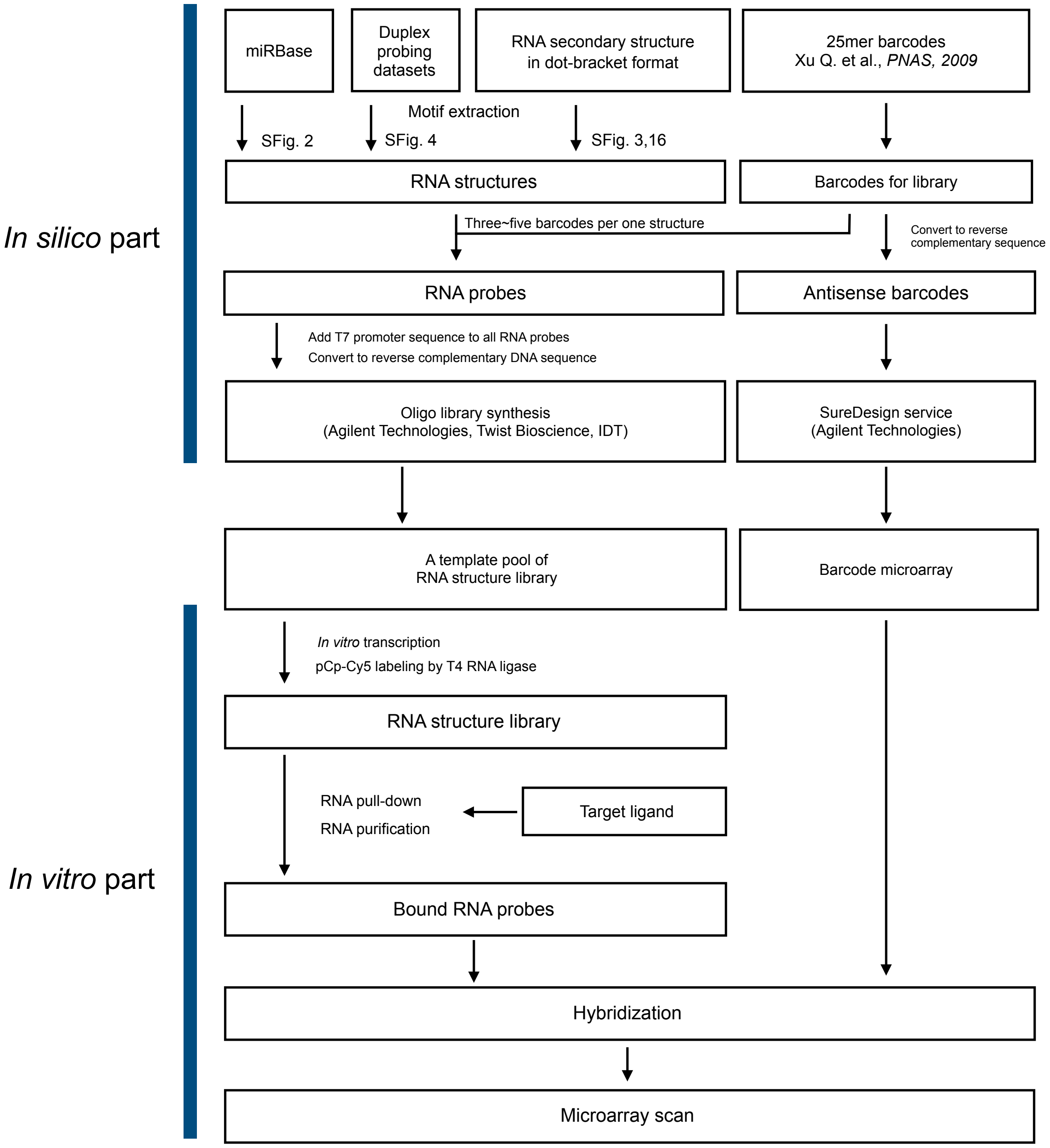

Supplementary Figure 1 | Experimental workflow of FOREST

FOREST is a system that examines specific interactions using an RNA structure library. An *in silico* pipeline designs the RNA structure library and barcode microarray for the downstream biochemical affinity assay. The bound RNAs are collected by an affinity enrichment assay and quantified by a microarray scan.

Supplementary Figure 1, Komatsu KR et al.

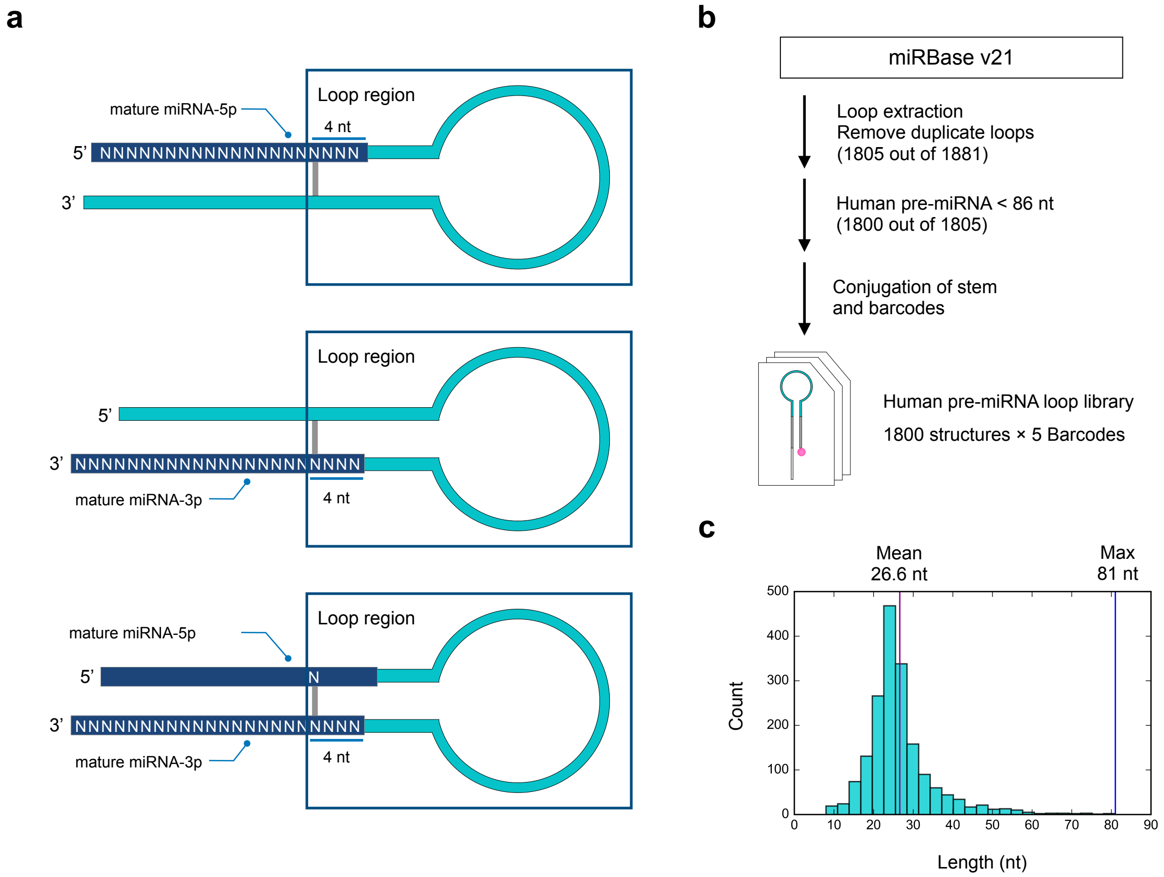

Supplementary Figure 2 | Design of pre-miRNA loop library

(**a**) The *in silico* extraction of loop motifs from human pre-miRNA. A pre-miRNA loop motif is defined as follows. (i) Start and end nucleotides should be in the same column on the secondary structure of the pre-miRNA presented in miRBase. (ii) If the pre-miRNA produces one mature miRNA, the start nucleotide of the loop region is set as the fourth nucleotide from the end on the loop-side of the mature miRNA sequence. (iii) If a pre-miRNA produces two mature miRNAs, the strand closer to the basal end is chosen, and the loop region is set as (ii).

(**b**) The design of the human pre-miRNA library from miRBase. Due to the synthetic limitation, we filtered pre-miRNA loops longer than 85 nt. The extracted loops were attached to the stem and barcode sequences.

(**c**) Histogram of loop lengths (nt). The purple line indicates the average, and the blue line indicates the maximum length.

Supplementary Figure 2, Komatsu KR et al.

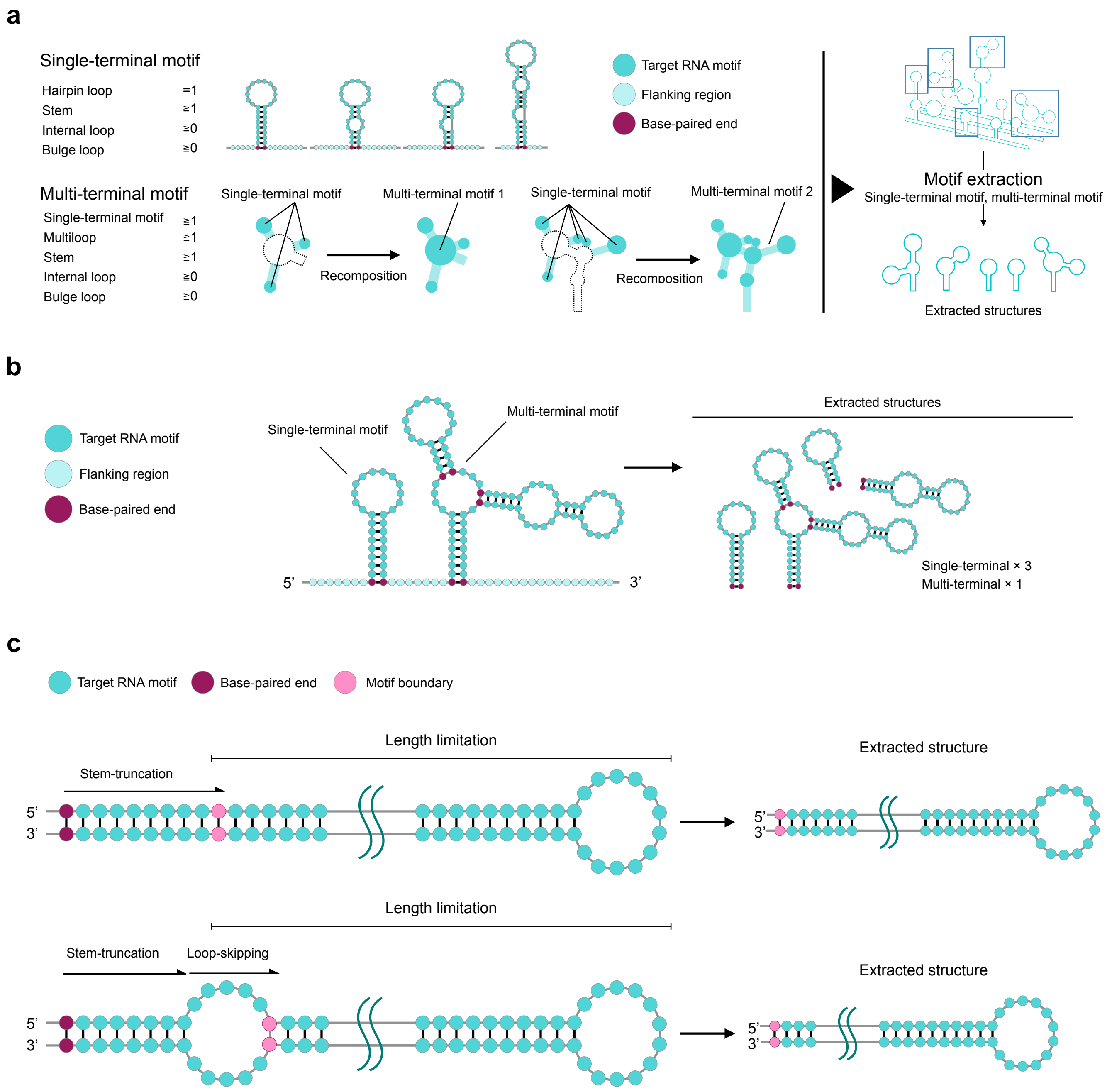

Supplementary Figure 3 | Definition of terminal motifs as target RNA structures

(**a**) Extraction of terminal motifs. A single-terminal motif is defined as a specific stem-loop motif consisting of a hairpin loop motif and adjacent double-stranded RNA stem. The RNA stem can include one or more bulge and/or internal loop structures. A multi-terminal motif consists of two or more single-terminal motifs with a multiloop.

(**b**) A representative example of a motif extraction in the case of terminal motifs. The target RNA motifs are highlighted by turquoise with the base-paired boundaries indicated in red. The adjacent region is highlighted in light blue.

(**c**) A structure-based truncation. If a target RNA motif is longer than the RNA length limitation, the motif will repeatedly shorten to the maximum length or the nearest base-paired end. The boundary of the motif is highlighted in pink. In this study, the limit is 116 nt due to the maximum length of the oligo DNA pool.

Supplementary Figure 3, Komatsu KR et al.

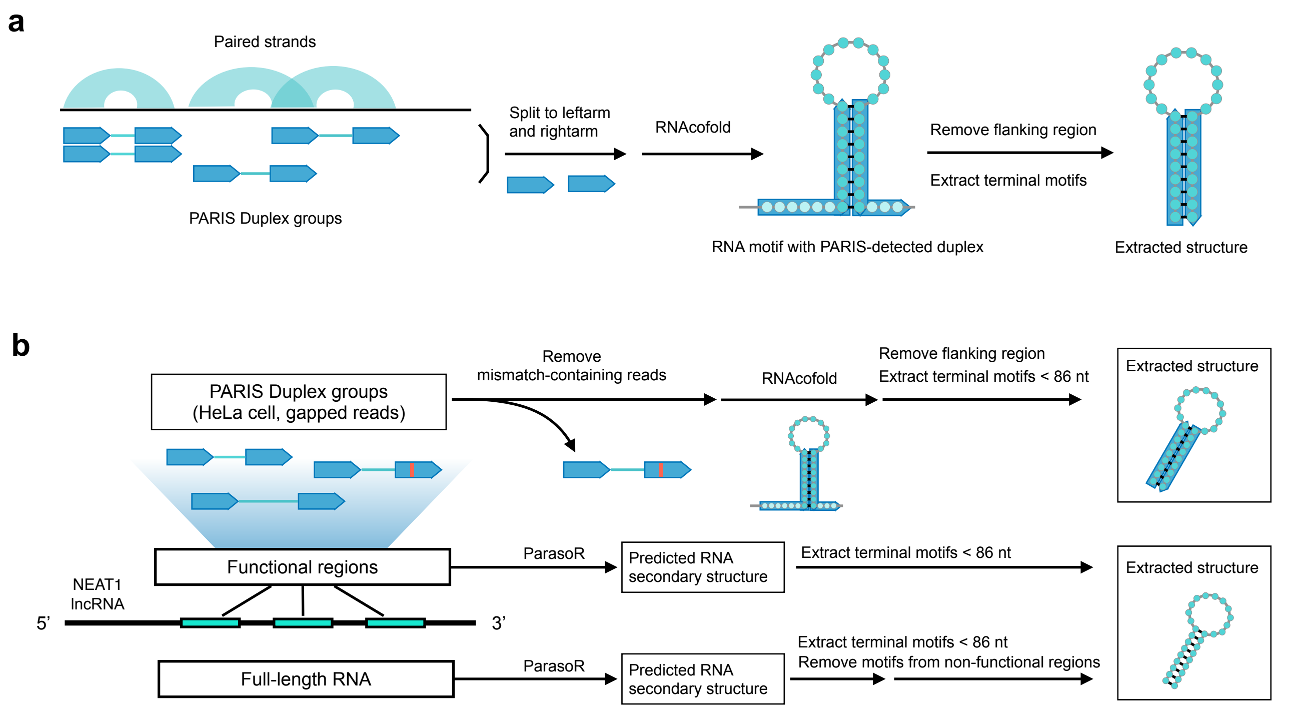

Supplementary Figure 4 | Motif extraction from duplex map datasets

(**a**) Schematic of the motif extraction using mapped duplex groups. The RNA secondary structure that has a validated stem structure was predicted by RNAcofold with default parameters and the input of a PARIS-detected duplexed group.

(**b**) Design of the human lncRNA NEAT1 library using the PARIS dataset and predicted structures. As a demonstration, we selected three functional regions of human NEAT1 lncRNA. We used PARIS duplex groups of HeLa cells and predicted secondary structures as the input. Extracted structures were filtered according to the length (nt) and genome location. In the case of RNA structure library, v1, the length limitation was set to 86 nt.

Supplementary Figure 4, Komatsu KR et al.

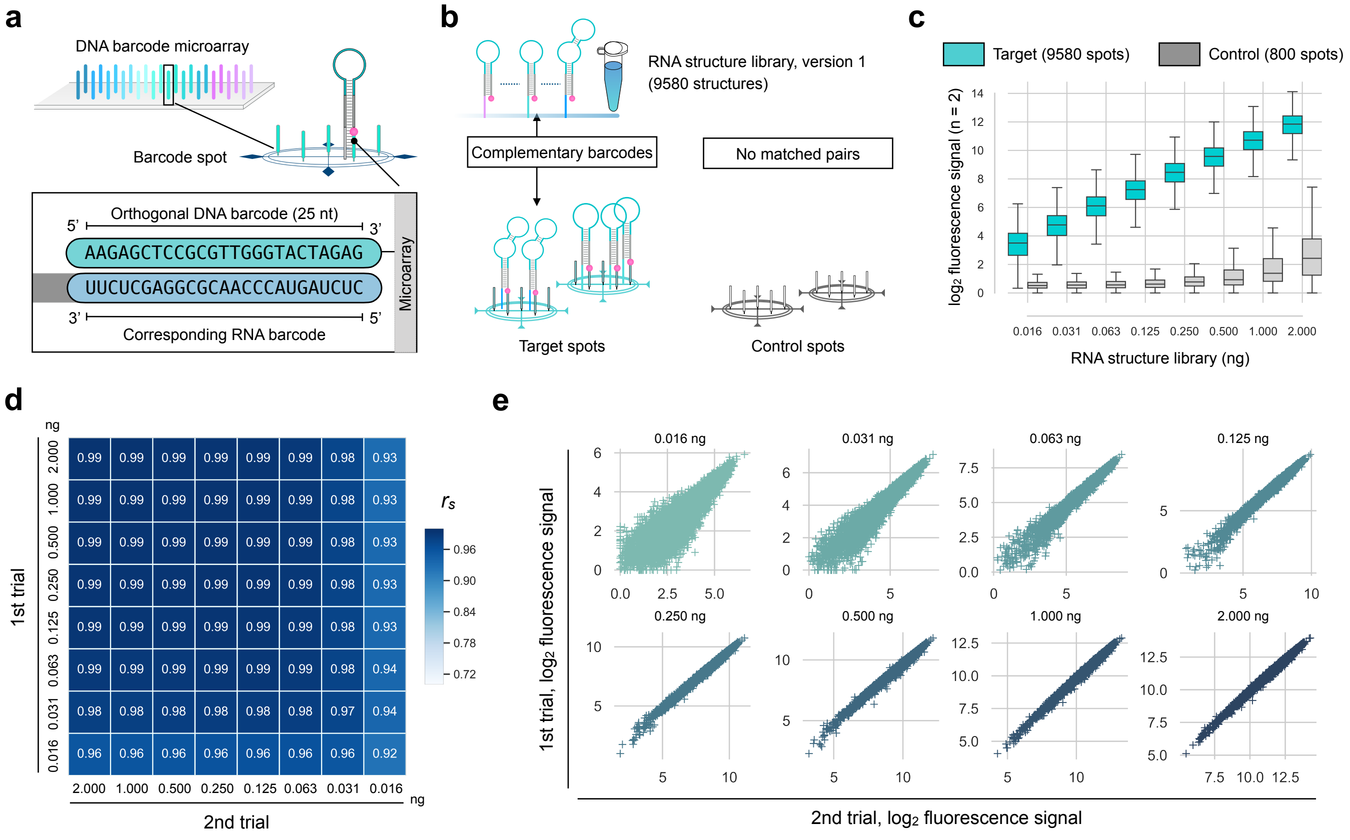

Supplementary Figure 5 | Fluorescence signal scanning on DNA barcode microarray

(**a**) Schematic of the barcode-based hybridization on a custom DNA microarray. Each spot on the microarray contains DNA sequences complementary to one barcode region of the RNA probe in the library.

(**b**) Schematic of the spot design. The microarray contains target spots that have complementary barcodes and controls spots that have barcodes that do not match any barcode in the library.

(**c**) A box plot of the fluorescence signals indicates high orthogonality and linearity. The intensities were determined by averaging two independent experiments. The box plot elements are defined as follows: center line, median; box limits, upper and lower quartiles; whiskers, 1.5x interquartile range. Green: target spots that have complementary barcodes (9580 spots). Gray: control spots that have barcodes that do not match any barcode in the library (800 spots).

(**d**) The matrix of Spearman's rank correlation coefficients calculated from the fluorescence signals of target spots (9580 spots) between two independent trials of the hybridization test.

(**e**) Scatter plots of the fluorescence signals (9580 spots) using various amounts of RNA.

Supplementary Figure 5, Komatsu KR et al.

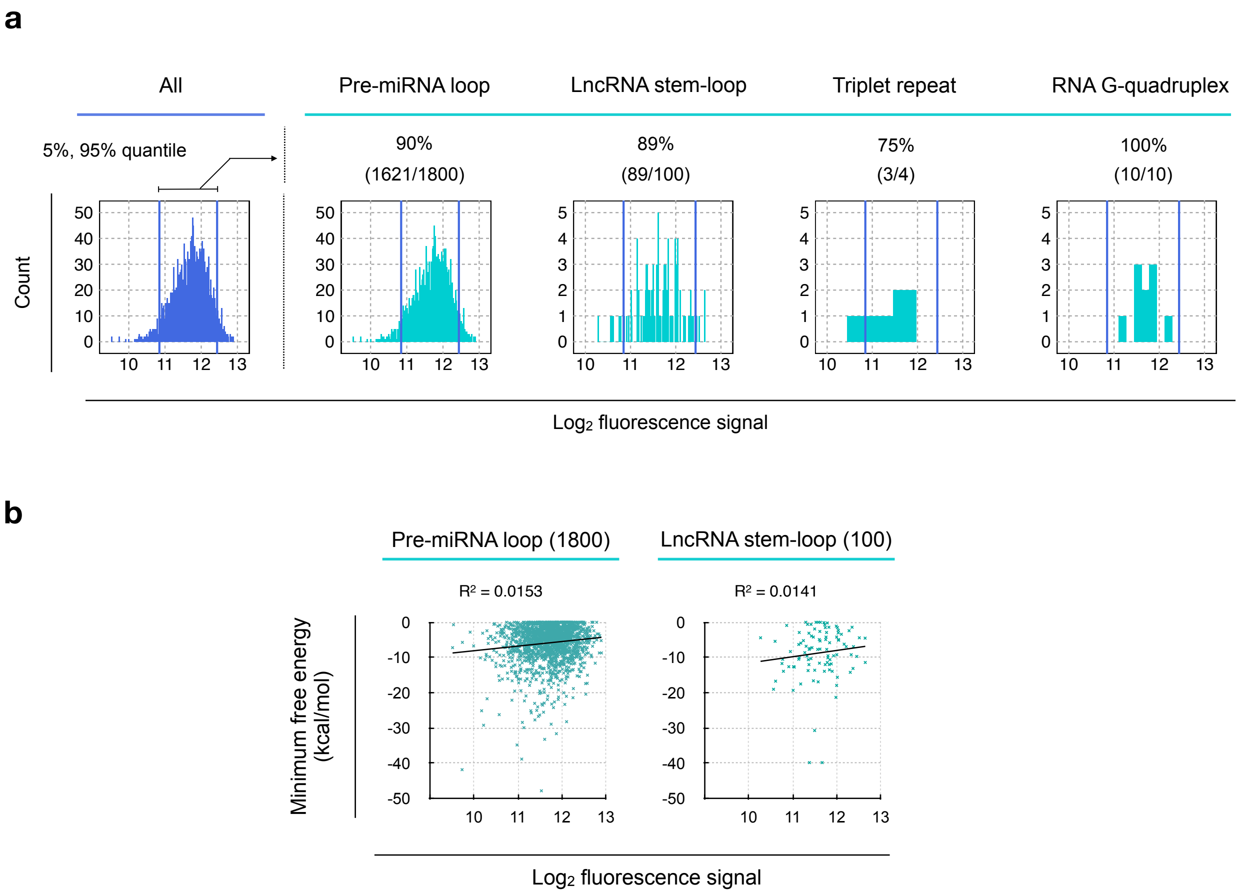

Supplementary Figure 6 | Confirmation of detection bias for each RNA class and RNA secondary structure

(**a**) Fluorescence intensities of each spot detected by a DNA barcode microarray are shown as histograms. Histograms of the fluorescence intensities are shown for all RNA probes in RNA structure library, v1 (1916 structures) and for each sub-library, where the x-axis is the fluorescence intensity and the y-axis is the data count. The purple vertical lines represent 5% and 95% quartiles of all RNA probes (1916 structures). The number and percentage of sub-libraries that fell within the 5-95% quartile are shown above the histograms. The fluorescence signals were calculated from the average of two independent experiments using a 1.0 ng RNA structure library.

(**b**) Scatter plots of the fluorescence intensity and minimum free energy of each spot detected by the DNA barcode microarray, where the x-axis is the fluorescence intensity and the y-axis is the minimum free energy (kcal/mol) of each RNA calculated by RNAfold. The fluorescence signals of pre-miRNA loops (1800 structures) and lncRNA motifs (100 structures) were calculated from the average of two independent experiments using a 1.0 ng RNA structure library. The black line represents a linear trend line.

Supplementary Figure 6, Komatsu KR et al.

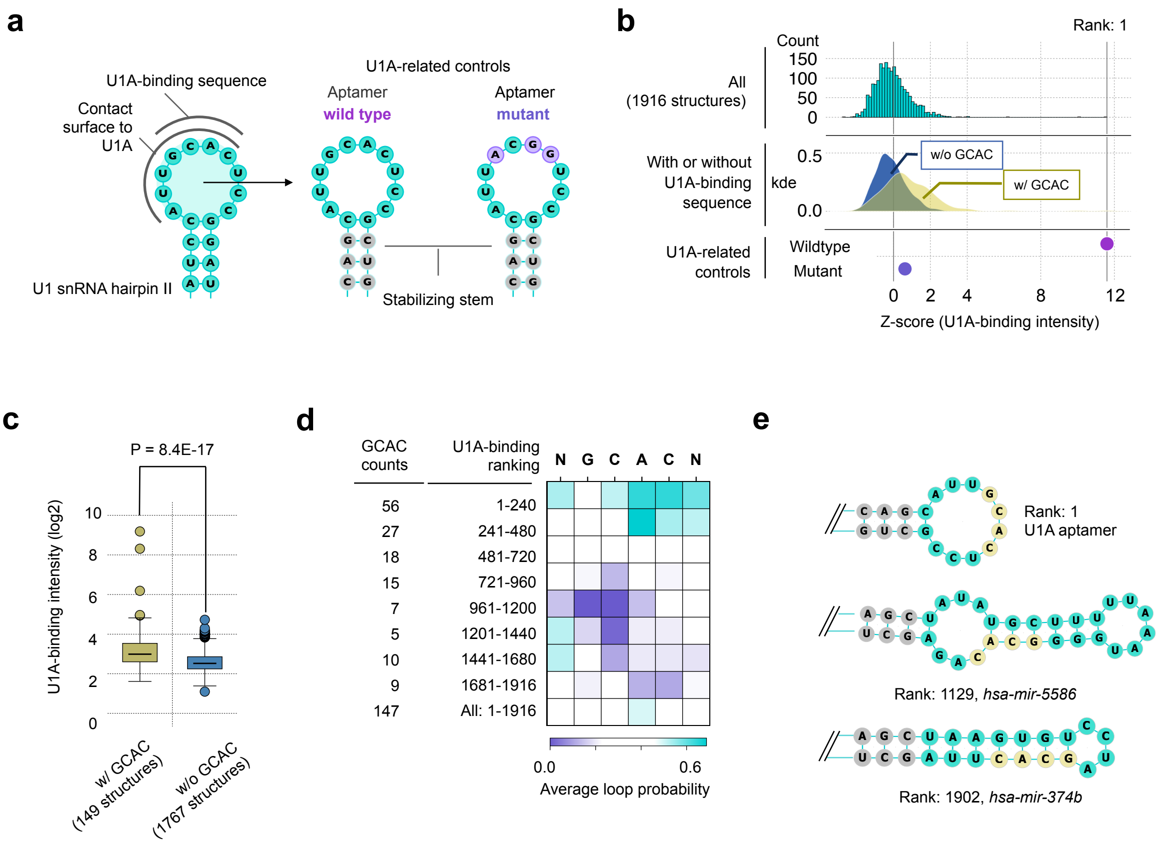

Supplementary Figure 7 | Validation of FOREST using U1A-binding RNA structures

(**a**) Design of the U1A-related controls in the library. U1 snRNA hairpin II was selected as the U1A-binding control. We also prepared its defective mutant by replacing three nucleotides (purple). The native stem region was replaced with a stabilizing stem (silver).

(**b**) Overview of the U1A-binding interactome. The data were calculated from the average of two independent experiments. A histogram representing all RNA structures in the library (top). Kernel density estimation of the U1A-binding intensities (middle). Yellow: RNA structures with GCAC, blue: RNA structures without GCAC. The point plot shows the U1A-related controls (bottom). Magenta: U1A aptamer wild type, purple: U1A aptamer mutant.

(**c**) Box plots of U1A-binding intensities indicate a significant difference between RNA structures with (149 structures) and without the GCAC sequence (1767 structures). The data were calculated from the average of two independent experiments. Box plot elements are defined as follows: center line, median; box limits, upper and lower quartiles; whiskers, 1.5x interquartile range; points, outliers. The p-value was determined by the two-tailed Brunner-Munzel test.

(**d**) Loop structure preference of the U1A-binding GCAC sequence. The loop probabilities were calculated with RNA structures that contain a single GCAC sequence (in total, 147 RNA structures were analyzed) and adjacent nucleotides at both the 5' and 3' ends (represented as "N"). The loop probabilities were averaged for every eight equal divisions (240 structures) and the total population of probes (1916 structures).

(**e**) Representative RNA structures with minimum free-energy structures. The GCAC sequences are highlighted in yellow. The stabilizing stem is highlighted in silver.

Supplementary Figure 7, Komatsu KR et al.

a

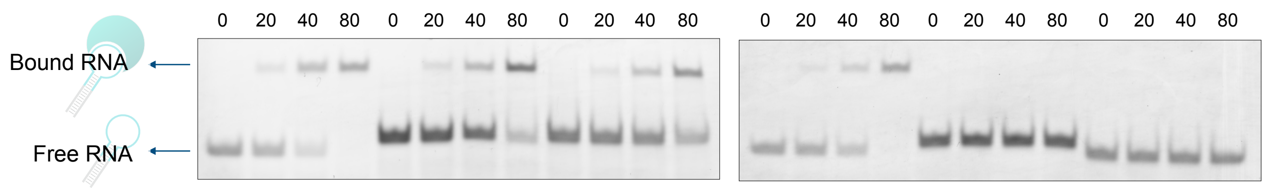

Structure:	U1A aptamer	*hsa-mir-3661*	*hsa-mir-6845*	U1A aptamer	*hsa-mir-374b*	U1A mutant
Rank:	1	2	3	1	1902	388
Z-score:	11.598	10.047	6.273	11.598	-1.858	0.618
Sequence:	AUUGCACUCC	CUUGCACUCG	CUUGCACACC	AUUGCACUCC	CUAGCACUUA	AUUACGGUCC
MFE structure:	LLLLLLLLLL	LLLSSLLLLS	LSSSLLLLLL	LLLLLLLLLL	LLLSSSSSSS	LLLLLLLLLL

b

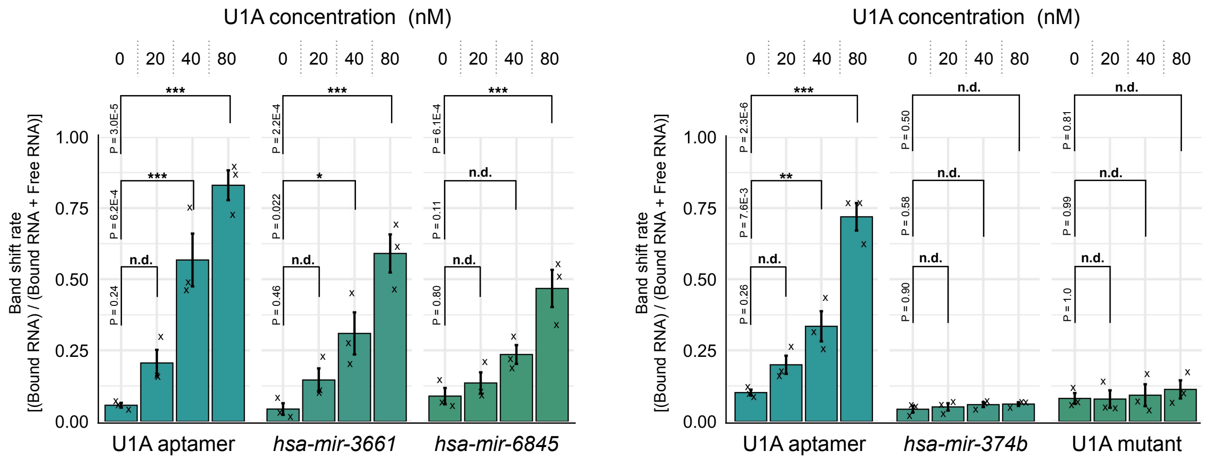

Supplementary Figure 8 | EMSA validation of U1A-binding RNA structures

(**a**) The gel image of an electrophoretic mobility shift assay using 50 nM RNA. U1A solutions were prepared by adjusting to 0 nM, 20 nM, 40 nM, and 80 nM. The table contains Z-scores, GCAC-centered sequences, and their minimum free-energy structures. “L” indicates a loop region and “S” indicates a stem region. The image shows representative data from three independent experiments. Source data are provided as a Source Data file.

(**b**) The bar plots represent the band shift ratios observed in (a). The error bars indicate means ± s.e.m. The experiments were performed with three biological replicates. The p-values were determined by two-tailed Dunnett’s test. ***p <0.001, **p < 0.01, *p < 0.05. n.d. indicates no significant difference. Each “x” indicates a data point.

Supplementary Figure 8, Komatsu KR et al.

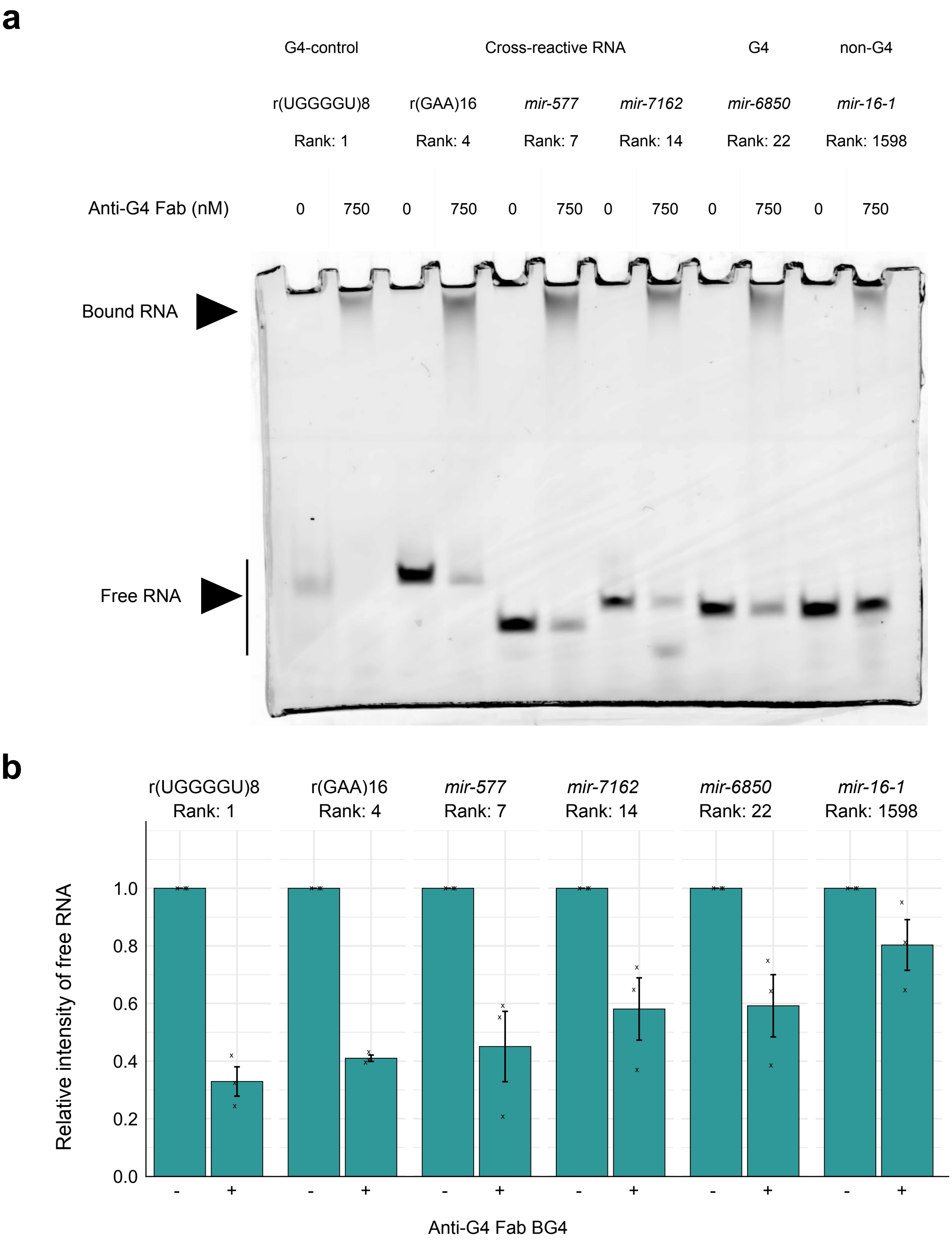

Supplementary Figure 9 | EMSA using cross-reactive RNAs with a Fab fragment of BG4 DNA/ RNA anti-G4 antibody

(**a**) A gel image of an electrophoretic mobility shift assay (EMSA) using 50 nM RNA. The RNP solution was prepared with 0 nM and 750 nM His-tagged BG4 anti-G4 antibody Fab fragment (Absolute Antibodies, Ab00174-1.6). The image shows representative data from three independent experiments. Source data are provided as a Source Data file.

(**b**) The bar plot represents the free RNA ratio observed in (a). The error bars indicate means ± s.e.m. The experiments were performed with three biological replicates. Each “x” indicates a data point.

Supplementary Figure 9, Komatsu KR et al.

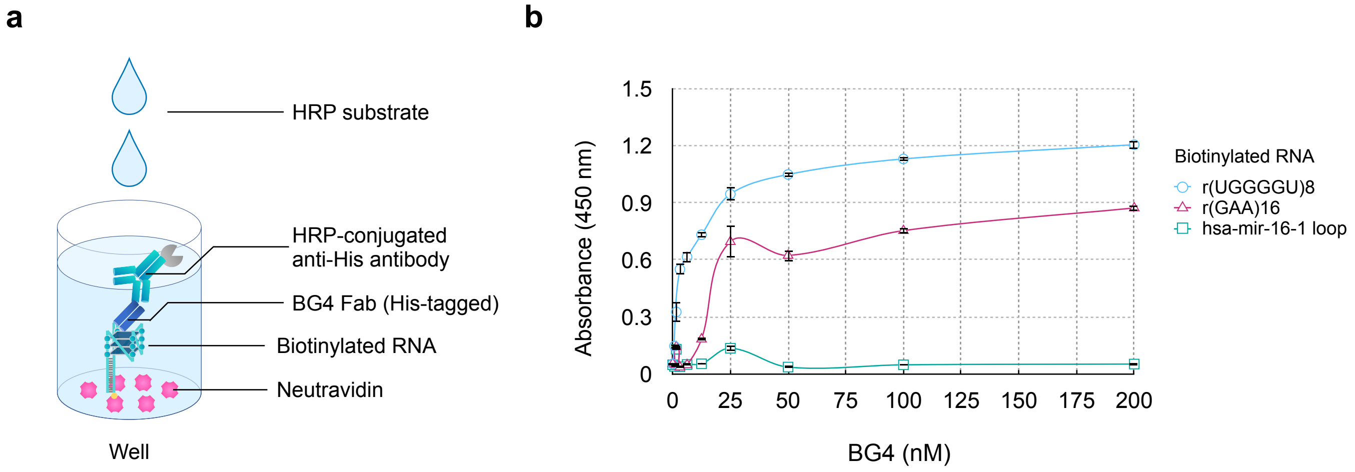

Supplementary Figure 10 | Validation of rG4 by ELISA

(**a**) Schematic of the enzyme-linked immunosorbent assay (ELISA). 100 nM biotinylated RNA and different concentrations of His-tagged BG4 Fab were mixed and incubated to form RNP complexes and immobilized on the surface of a neutravidin-coated well. Then, HRP-labeled anti-6xhistidine antibodies were added to recognize BG4 and enzymatically process the HRP substrate. Finally, the activities of well-bound HRP were quantitatively measured as the absorbance of processed HRP substrate (TMB, 3,3’,5,5’-tetramethylbenzidine). See also Supplementary Methods.

(**b**) The dots and lines represent the absorbance of r(UGGGGU)8, r(GAA)16, and the *hsa-mir-16-1* loop. The y-axis indicates the absorbance of TMB (450 nm), and the x-axis indicates the concentration of BG4. The error bars indicate means ± s.e.m.; the ELISA were performed with three replicates. Source data are provided as a Source Data file.

Supplementary Figure 10, Komatsu KR et al.

a

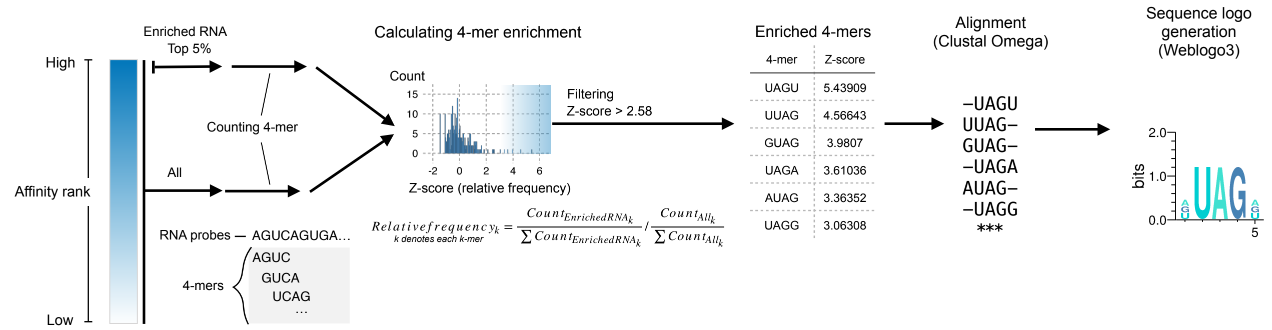

$$Relative frequency_k = \frac{Count_{EnrichedRNA_k}}{\sum Count_{EnrichedRNA_k}} / \frac{Count_{All_k}}{\sum Count_{All_k}}$$

b

Enriched 4-mer(s)

U1A 4-mer	Z-score
GCAC	5.665
CACA	5.47463
ACAC	4.59281
UGCA	2.62993

LIN28A 4-mer	Z-score
GAUA	5.44064
AUCG	2.64811
GGAG	2.58044

DHX36 4-mer	Z-score
GGGG	6.17285
GGGA	3.68403
GGGC	3.40954
AGGG	3.33962
UGGG	3.14941
GGCG	2.80215

CIRBP 4-mer	Z-score
GGGG	4.28524
GGUU	3.23921
CAUA	2.91703
GGAU	2.67609
AGGG	2.60933

BG4 4-mer	Z-score
AGGG	3.47015
CGAU	2.99295
GAUA	2.93459
AUAG	2.61287

Logo

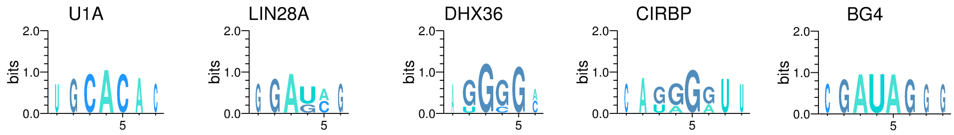

Supplementary Figure 11 | Sequence motif analysis with FOREST datasets

(**a**) Schematic of the sequence motif logo generation. Alignment of the enriched 4-mers was performed by the Clustal Omega website. Sequence logo generation was performed by the Weblogo3 website.

(**b**) Enriched 4-mers and sequence logos of the top 5% enriched sequence motifs for each protein. The top 5% of pre-miRNA loops (1800 structures) was extracted and analyzed.

Supplementary Figure 11, Komatsu KR et al.

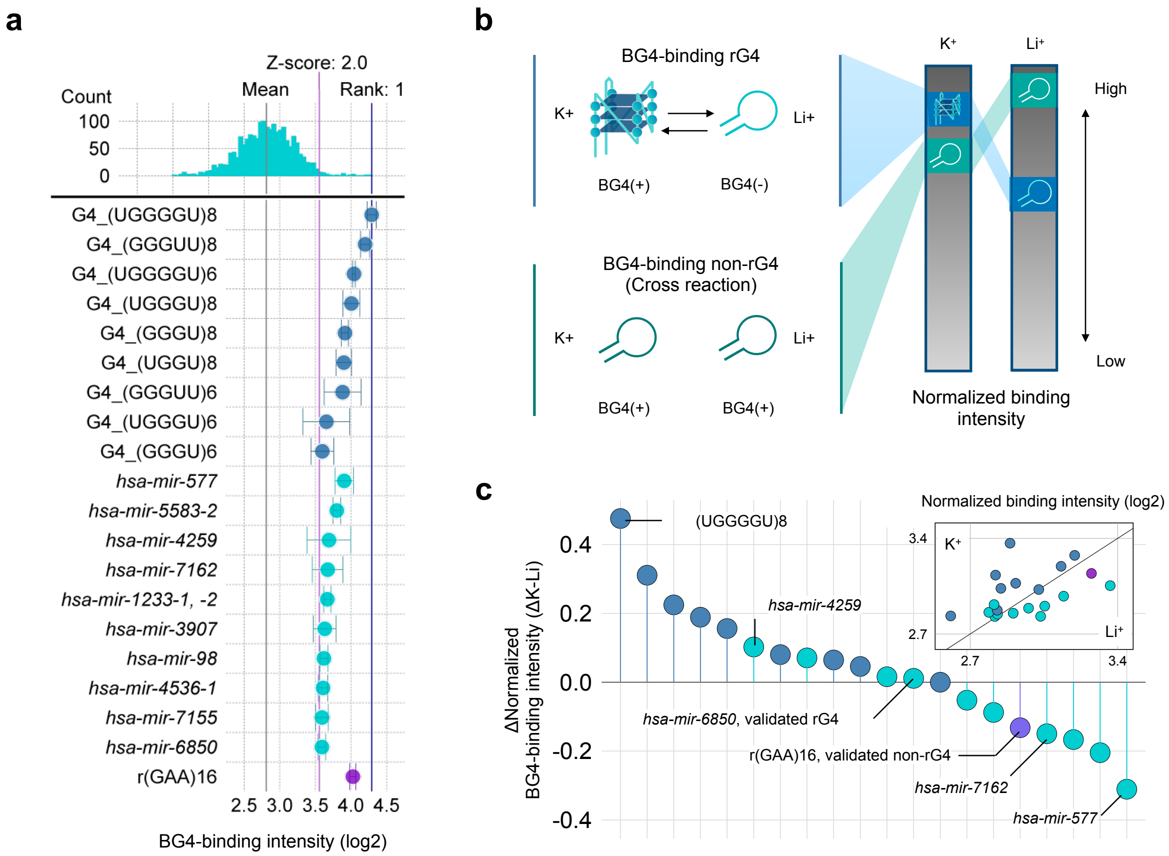

Supplementary Figure 12 | Evaluation of BG4-binding properties under different conditions in rG4 stabilization

(**a**) A histogram of the averaged BG4-binding intensities calculated from two independent experiments. The point plot below shows the binding intensity of representative pre-miRNAs (turquoise), rG4 controls (blue), and triplet repeats (magenta) from the structures with the top 20 BG4-binding intensities. The error bars indicate means ± s.d. of multiple barcodes assigned to each RNA. The vertical lines indicate the mean (gray), Z-score = 2.0 (purple), and top score (navy).

(**b**) Schematic of the binding properties of BG4. Monovalent cations affect the formation and stability of rG4, leading to dynamic changes in the binding intensity for BG4.

(**c**) The point plot shows the normalized binding intensities of the high-affinity structures shown in (a) and their differences (ΔK-Li) between potassium and lithium conditions.

Supplementary Figure 12, Komatsu KR et al.

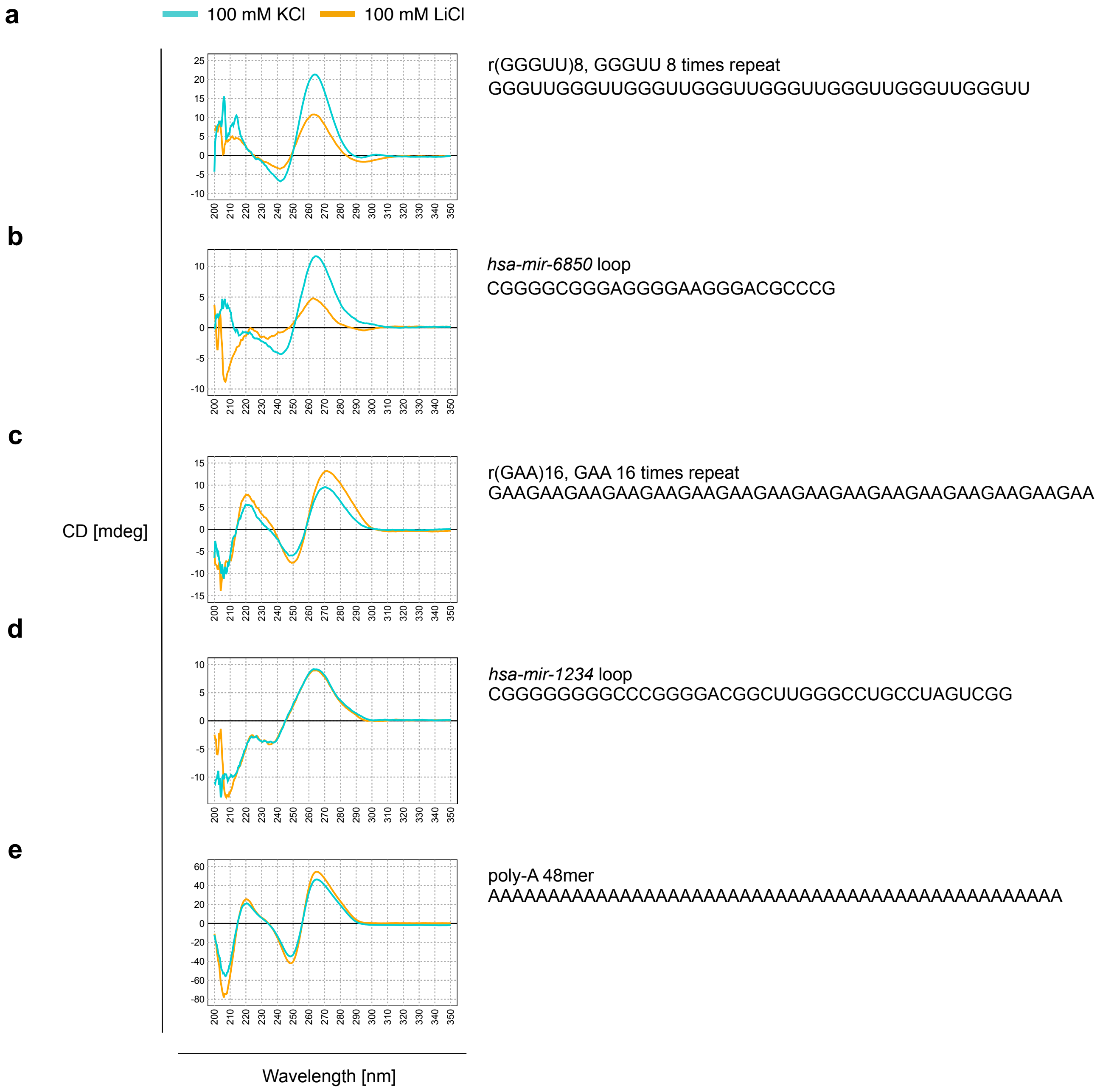

Supplementary Figure 13 | Validation of RNA structures by circular dichroism (CD) analysis of RNA

(**a-e**) A CD spectrum analysis of 1 μM RNA in the presence of 100 mM KCl or 100 mM LiCl. The spectra of RNA G4 show a negative peak at 241 nm and a positive peak at 265 nm. These rG4-specific peaks were diminished in the presence of lithium ion because the probability of rG4 is higher in the presence of potassium ions than of smaller cationic ions.
(**a**) RG4 control, GGGUU repeat. (**b**) *hsa-mir-6850* loop. (**c**) GAA repeat. (**d**) *hsa-mir-1234* loop. (**e**) Non-rG4 control, poly-A RNA.

Supplementary Figure 13, Komatsu KR et al.

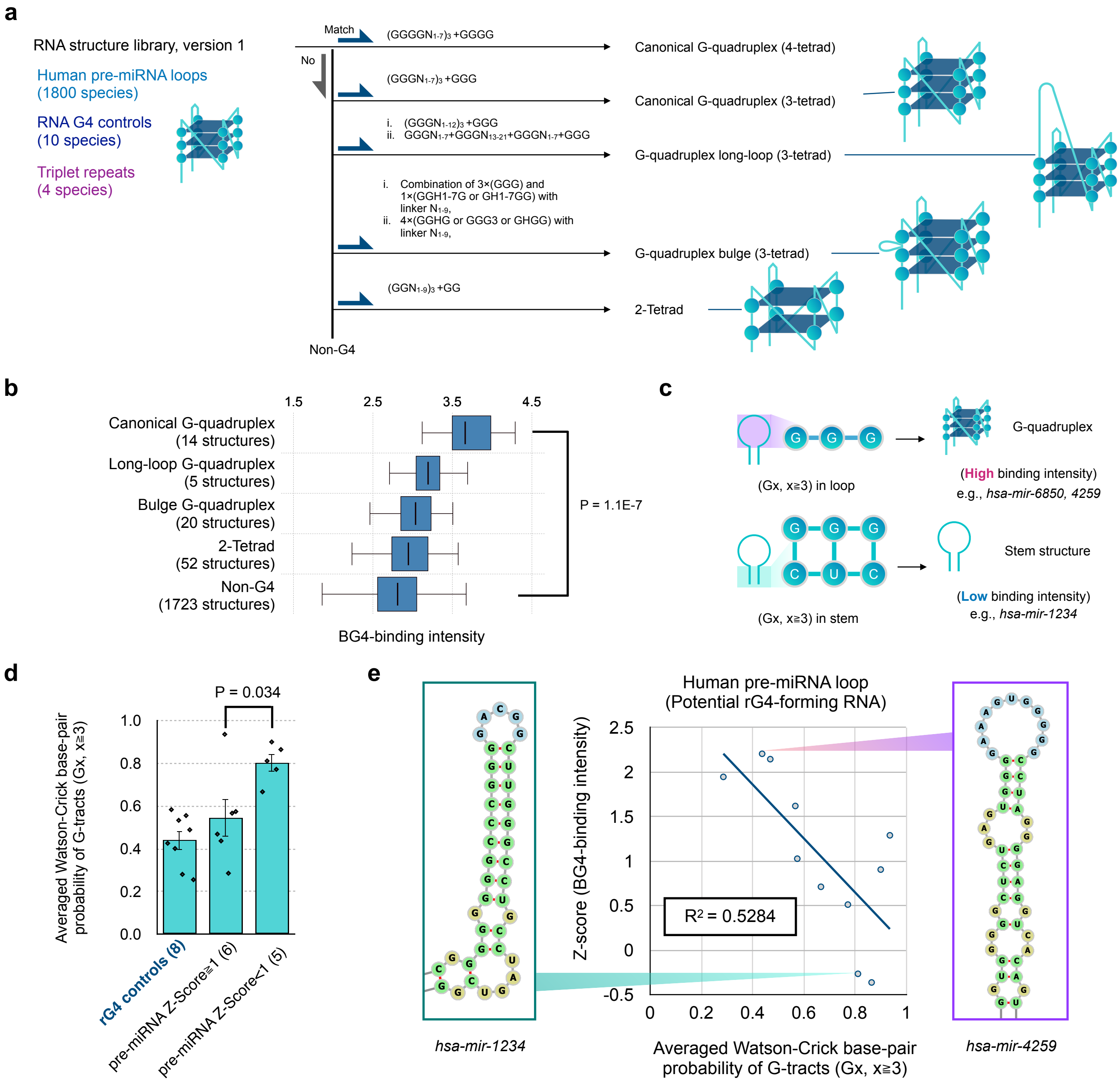

Supplementary Figure 14 | Sequence-based classification of RNA G-quadruplex and relation to BG4-binding intensities

(**a**) A flowchart of the sequence-based rG4 classification. For example, the definition of a three-layered canonical rG4 is $GGGN_{1\text{-}7}GGGN_{1\text{-}7}GGGN_{1\text{-}7}GGG$. N stands for any nucleotide A, U, G, or C. H stands for A, U, or C.

(**b**) A box plot showing BG4-binding intensities for each rG4 category. The numbers of probes are shown in parentheses. The averaged binding intensity of canonical rG4 was significantly higher than that of non-rG4. The average BG4-binding intensity correlated with the stability of each rG4 (Canonical > Long > Bulge > 2-tetrad). Box plot elements are defined as follows: center line, median; box limits, upper and lower quartiles; whiskers, 1.5x interquartile range. The p-value was determined by the two-tailed Brunner-Munzel test.

(**c**) Schematic of the base-pair equilibrium on rG4-forming RNA. The probability of a Watson-Crick base pair is an essential factor for the formation of rG4 because Watson-Crick base pairing competes with Hoogsteen base pairing, which is needed for rG4 folding.

(**d**) The average Watson-Crick base-pair probability of potential rG4-forming pre-miRNA that possesses four repeats of G-tracts (Gx, x≧3, total 11 structures). Although the probability of the binding population (Z-score of BG4-binding intensity ≧ 1.0) was similar to that of the rG4 controls, the nonbinding RNA structures had a high probability of Watson-Crick base pairs. The error bars indicate means ± s.e.m., and the p-value was determined by a two-tailed t-test. The number of RNA structures is shown in parentheses. Each "◇" indicates a data point.

(**e**) Watson-Crick base pairing influences BG4-binding intensity. The BG4-binding intensity of potential rG4-forming pre-miRNA was negatively correlated with Watson-Crick base-pair probability. Representative minimum free-energy structures are represented in the green and purple boxes.

Supplementary Figure 14, Komatsu KR et al.

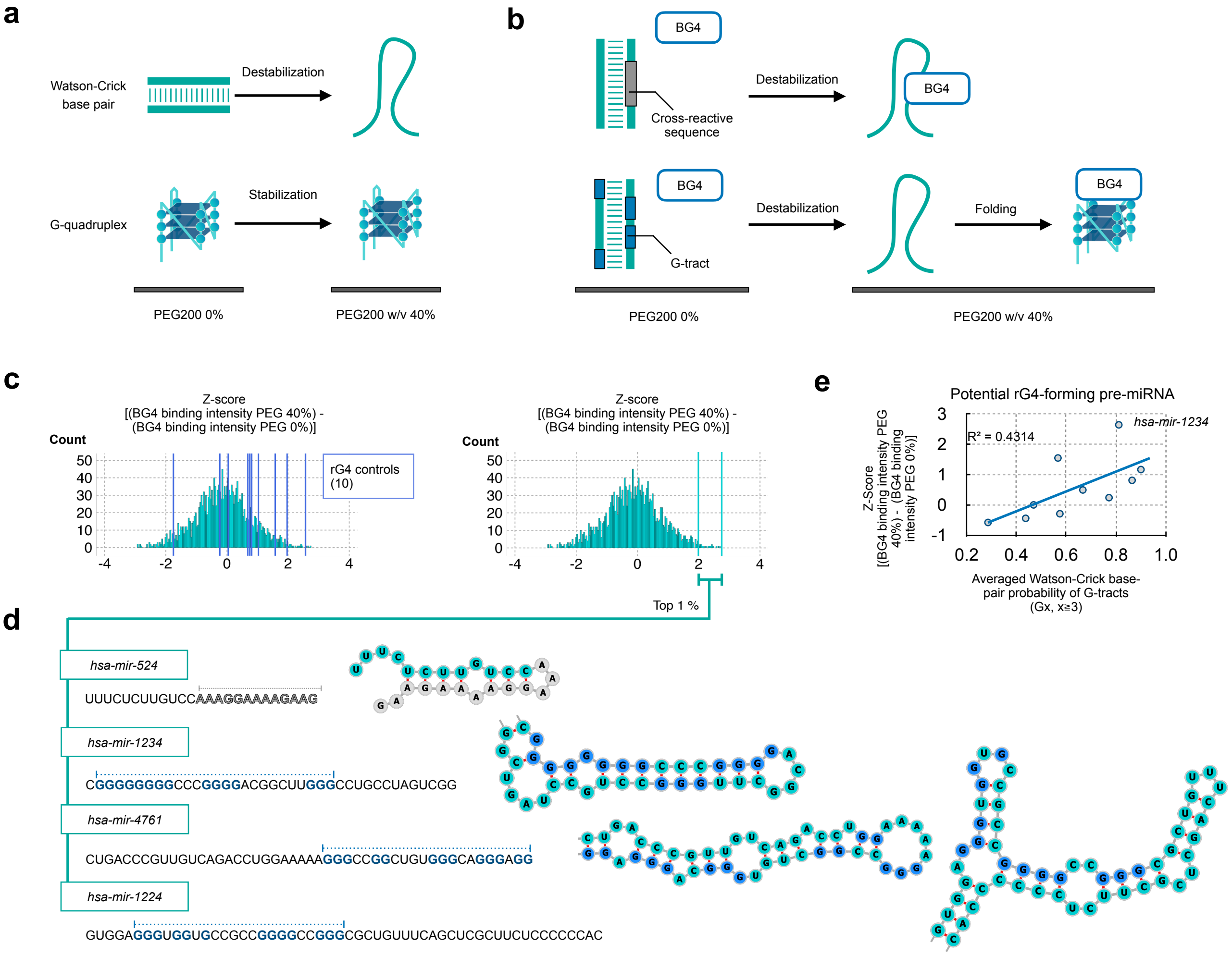

Supplementary Figure 15 | Functional remodeling of RNA structures in a crowded environment with small molecules

(**a**) Schematic of dynamic RNA structures affected by PEG200 (with an average molecular weight ≒ 200 g/mol) w/v 40%. Watson-crick base-pairing was destabilized, whereas G-quadruplex was stabilized.

(**b**) Schematic of BG4-RNA interactions affected by PEG200. The interaction between BG4 cross-reactive RNA and rG4 is expected to strengthen because of structural rearrangement triggered by PEG200.

(**c**) Histograms of BG4-binding differences with annotation to rG4 controls (10 structures, blue lines) and the top 1% of binding differences (green lines) in RNA structure library, v1 (1916 structures). Eight of the rG4 controls have higher binding intensities under the PEG200 condition, suggesting rG4 were more stabilized and reacted to BG4 by PEG200. To mimic the cellular condition, we analyzed RNP interactions under phosphate buffer (pH 7.0, 140 mM KCl, 10 mM NaCl).

(**d**) Representative minimum free-energy structures. These structures possess the base-paired G-tracts and cross-reactive sequence (AG-tract) to BG4. Like the model in (b), PEG200 may loosen these stem structures, exposing the binding site to BG4 or assisting the formation of the G-quadruplex structure.

(**e**) The correlation between the average Watson-Crick base-pair probability of potential rG4-forming pre-miRNA loops that possess four repeats of G-tracts (Gx, x≧3, total 11 structures) and BG4-binding differences.

Supplementary Figure 15, Komatsu KR et al.

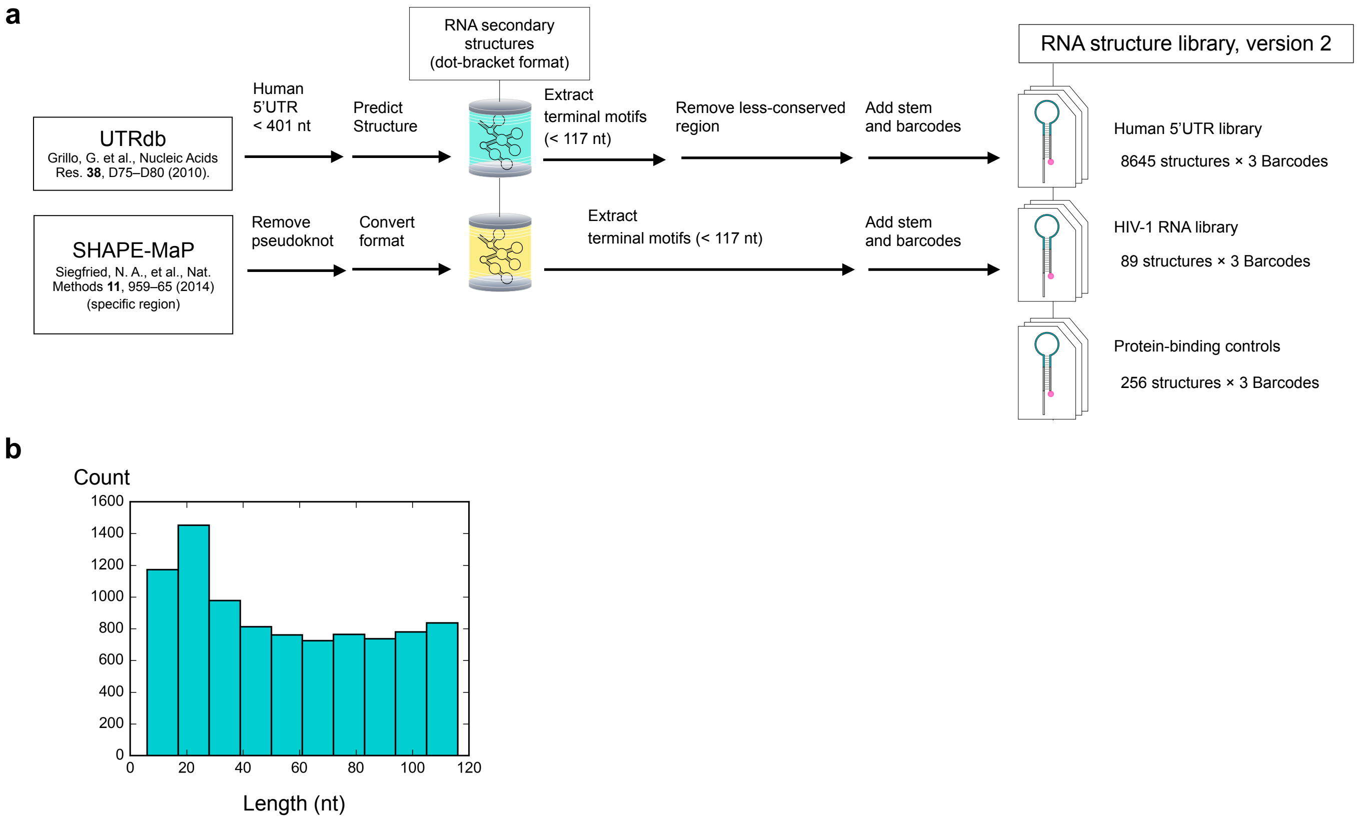

Supplementary Figure 16 | Design of human 5'UTR and HIV-1 library

(**a**) *In silico* extraction of terminal motifs from large-scale datasets of RNA secondary structures for the construction of RNA structure library, v2.

(**b**) Histogram of the RNA motif length (nt) in RNA structure library, v2, derived from UTRdb and SHAPE-MaP (median: 52 nt, mean: 56 nt).

Supplementary Figure 16, Komatsu KR et al.

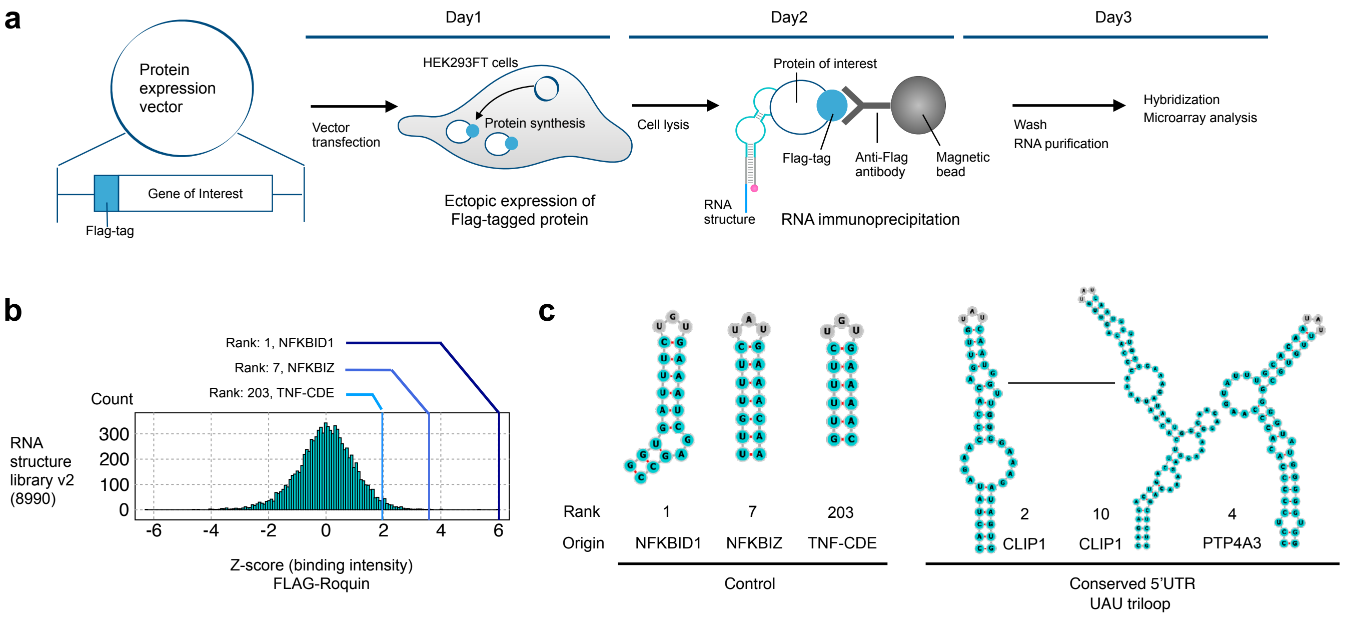

Supplementary Figure 17 | A rapid and general method using cell lysate and Flag-tags

(**a**) Schematic of RNA immunoprecipitation using cell lysates containing over-expressed Flag-tagged proteins. HEK293FT cells were transfected with an RNA-binding protein expression vector. Next, the cell lysate-derived Flag-tagged protein was immobilized on magnetic beads and interacted with the RNA structure library. The RNA structures were collected the next day and quantified on a microarray.

(**b**) A histogram of Z-scores of the Roquin-binding intensity. RNA immunoprecipitation used RNA structure library, v2, and HEK293FT cell lysates containing Flag-tagged Roquin. The x-axis represents the Z-score of the Roquin binding intensity, and the y-axis represents frequency. The vertical lines indicate the Z-score of positive controls known to bind Roquin.

(**c**) Representative Roquin-binding RNA secondary structures. Each structure is shown with the rank of the binding score in RNA structure library, v2 (8990 structures), and the original gene symbol. U-R-U triloop motifs (R = A or G) that are essential for the interaction with Roquin are shown in gray. Rank-10 is a multi-terminal loop motif that includes Rank-2.

Supplementary Figure 17, Komatsu KR et al.

Supplementary Table 1. RNA probe sequences related to LIN28A or U1A

ID	Name	Annotation	RNA sequence (5'-3')	Purpose	Figure	Method
LIN28A-01	hsa-mir-98	hsa-mir-98 loop	GGUGUACGAAGUUUCAGCAUUGUUG<br>UGGGGUAGGGAUAUUAGGCCCCAAU<br>UAGAAGAUAACUAUGCUGAAGCUUC<br>GUGCACC	EMSA	Fig. 4	*in vitro* transcription
LIN28A-02	hsa-let-7a-3	hsa-let-7a-3 loop	GGUGUACGAAGUUUCAGCAUAGUUU<br>GGGGCUCUGCCCUGCUAUGGGAUAA<br>CUAUGCUGAAGCUUCGUGCACC	EMSA	Fig. 4	*in vitro* transcription
LIN28A-03	hsa-let-7f-2	hsa-let-7f-2 loop	GGUGUACGAAGUUUCAGCAUAGUUU<br>UAGGGUCAUACCCCAUCUUGGAGAU<br>AACUAUGCUGAAGCUUCGUGCACC	EMSA	Fig. 4	*in vitro* transcription
U1A-01	U1A aptamer	U1A-related controls	GGUGUACGAAGUUUCAGCAUUGCAC<br>UCCGCUGAAGCUUCGUGCACC	EMSA	Supplementary Fig. 8	*in vitro* transcription
U1A-02	hsa-mir-3661	hsa-mir-3661 loop	GGUGUACGAAGUUUCAGCGCUGCUU<br>GCACUCGUUCAGCGCUGAAGCUUCG<br>UGCACC	EMSA	Supplementary Fig. 8	*in vitro* transcription
U1A-03	hsa-mir-6845	hsa-mir-6845 loop	GGUGUACGAAGUUUCAGCGAGCCCU<br>UGCACACCACCAGCCUCGCUGAAGC<br>UUCGUGCACC	EMSA	Supplementary Fig. 8	*in vitro* transcription
U1A-04	hsa-mir-374b	hsa-mir-374b loop	GGUGUACGAAGUUUCAGCUAAGUGU<br>CCUAGCACUUAGCUGAAGCUUCGUG<br>CACC	EMSA	Supplementary Fig. 8	*in vitro* transcription
U1A-05	U1A mutant	U1A-related controls	GGUGUACGAAGUUUCAGCAUUACGG<br>UCCGCUGAAGCUUCGUGCACC	EMSA	Supplementary Fig. 8	*in vitro* transcription

Supplementary Table 2. RNA probe sequences related to rG4-binding proteins

ID	Name	Annotation	RNA sequence (5'-3')	Purpose	Figure	Method
RG4-01	r(UGGGGU)8	G4 control	GGUGUACGAAGUUUCAGCUGGGGUUGGGGUUGGGGUUGGGGUUGGGGUUGGGGUUGGGGUUGGGGUGCUGAAGCUUCGUGCACC	EMSA, NMM assay, ELISA	Fig. 5 and Supplementary Figs. 9, 10	*in vitro* transcription
RG4-02	r(UGGGU)6	G4 control	GGUGUACGAAGUUUCAGCUGGGUUGGGUUGGGUUGGGUUGGGUUGGGUGCUGAAGCUUCGUGCACC	NMM assay	Fig. 5	*in vitro* transcription
RG4-03	hsa-mir-4259	hsa-mir-4259 loop	GGUGUACGAAGUUUCAGCGGUGGGGGCUCUGAGUGGGGAAAGUGGGGGCCUAGGGGAGGUCACAGUGCUGAAGCUUCGUGCACC	NMM assay	Fig. 5	*in vitro* transcription
RG4-04	hsa-mir-4296	hsa-mir-4296 loop	GGUGUACGAAGUUUCAGCCUCAGAGGGCUGAAGUGGUUGUGGGGAGGGGCUUCUGGGGCUGAAGCUUCGUGCACC	NMM assay	Fig. 5	*in vitro* transcription
RG4-05	hsa-mir-4516	hsa-mir-4516 loop	GGUGUACGAAGUUUCAGCGGGCAGGGAGGGCAGGGCAGGCUCUGGGGUGGGGGGUCUGUGAGUCAGCCACGGCUCGCUGAAGCUUCGUGCACC	NMM assay	Fig. 5	*in vitro* transcription
RG4-06	hsa-mir-6850	hsa-mir-6850 loop	GGUGUACGAAGUUUCAGCCGGGGCGGGAGGGGAAGGGACGCCCGGCUGAAGCUUCGUGCACC	EMSA, NMM assay	Fig. 5 and Supplementary Fig. 9	*in vitro* transcription
RG4-07	r(GAA)16	Triplet repeat	GGUGUACGAAGUUUCAGCGAAGAAGAAGAAGAAGAAGAAGAAGAAGAAGAAGAAGAAGAAGAAGAAGCUGAAGCUUCGUGCACC	EMSA, NMM assay, ELISA	Fig. 5 and Supplementary Figs. 9, 10	*in vitro* transcription
RG4-08	hsa-mir-577	hsa-mir-577 loop	GGUGUACGAAGUUUCAGCCCUGAUGAAUCUGAGGCCAGGGCUGAAGCUUCGUGCACC	EMSA, NMM assay	Fig. 5 and Supplementary Fig. 9	*in vitro* transcription
RG4-09	hsa-mir-7162	hsa-mir-7162 loop	GGUGUACGAAGUUUCAGCGCUGAACCCCUGCCCCUGGAGCCCCCAGCAGGGCCCUCGCUGAAGCUUCGUGCACC	EMSA, NMM assay	Fig. 5 and Supplementary Fig. 9	*in vitro* transcription
RG4-10	hsa-mir-1234	hsa-mir-1234 loop	GGUGUACGAAGUUUCAGCCGGGGGGGGCCCGGGGACGGCUUGGGCCUGCCUAGUCGGGCUGAAGCUUCGUGCACC	NMM assay	Fig. 5	*in vitro* transcription
RG4-11	hsa-mir-16-2	hsa-mir-16-2 loop	GGUGUACGAAGUUUCAGCUUGGCGUAGUGAAAUAUAUAUUAAACACCAAGCUGAAGCUUCGUGCACC	NMM assay	Fig. 5	*in vitro* transcription
RG4-12	hsa-mir-16-1	hsa-mir-16-1 loop	GGUGUACGAAGUUUCAGCUUGGCGUUAAGAUUCUAAAAUUAUCUCCAGGCUGAAGCUUCGUGCACC	EMSA, ELISA, NMM assay,	Fig. 5 and Supplementary Figs. 9, 10	*in vitro* transcription
RG4-13	hsa-mir-21	hsa-mir-21 loop	GGUGUACGAAGUUUCAGCGUUGACUGUUGAAUCUCAUGGCAACGCUGAAGCUUCGUGCACC	NMM assay	Fig. 5	*in vitro* transcription
RG4-14	r(GGGUU)8	G4 control	GGGUUGGGUUGGGUUGGGUUGGGUUGGGUUGGGUUGGGUU	CD analysis	Supplementary Fig. 13	Synthesized and purchased from GeneDesign (Osaka, Japan)
RG4-15	r(GAA)16	Triplet repeat	GAAGAAGAAGAAGAAGAAGAAGAAGAAGAAGAAGAAGAAGAAGAAGAA	CD analysis	Supplementary Fig. 13	Synthesized and purchased from GeneDesign (Osaka, Japan)
RG4-16	hsa-mir-6850	hsa-mir-6850 loop	CGGGGCGGGAGGGGAAGGGACGCCCG	CD analysis	Supplementary Fig. 13	Synthesized and purchased from GeneDesign (Osaka, Japan)
RG4-17	poly-A 48mer	non-G4 control	AAAAAAAAAAAAAAAAAAAAAAAAAAAAAAAAAAAAAAAAAAAAAAAA	CD analysis	Supplementary Fig. 13	Synthesized and purchased from GeneDesign (Osaka, Japan)
RG4-18	hsa-mir-1234	hsa-mir-1234 loop	CGGGGGGGGCCCGGGGACGGCUUGGGCCUGCCUAGUCGG	CD analysis	Supplementary Fig. 13	Synthesized and purchased from GeneDesign (Osaka, Japan)

Komatsu KR et al.

Supplementary Table 3. RNA probe sequences related to Fig. 6

ID	Name	Annotation	RNA sequence (5'-3')	Purpose	Figure	Method
EIF3-01	Rank-9 (EIF3)	EHMT1	GGAUACUAGCGCGGCUGUGAGUCUG<br>GGGUGUACGAGCGGGCGGGGCGAUG<br>GCGCGCGGGAGGGGCGGGGCCACGC<br>UGCGGGCCCGCUCGUGCACC	Pull-down assay	Fig. 6	*in vitro* transcription
EIF3-02	Rank-8910 (EIF3)	KIAA1299	GGAUACUAGCGCGGCUGUGAGUCUG<br>GGGUGUACGAGCAGGUGUUUGGACU<br>AACCUGCUCGUGCACC	Pull-down assay	Fig. 6	*in vitro* transcription
EIF3-03	Rank-13 (EIF3)	HIV-1 gag IRES	GGAUACUAGCGCGGCUGUGAGUCUG<br>GGGUGUACGAGGGCCAGGGGGAAAG<br>AAACAAUAUAAACUAAAACAUAUAG<br>UAUGGGCAAGCAGGGAGCUAGAACG<br>AUUCGCAGUUAAUCCUGGCCCUCGU<br>GCACC	Pull-down assay	Fig. 6	*in vitro* transcription
EIF3-04	Rank-18 (EIF3)	HIV-1	GGAUACUAGCGCGGCUGUGAGUCUG<br>GGGUGUACGAGGCCAUAAAGCAAGA<br>GUUUUGGCUGAAGCAAUGAGCCAAG<br>UAACAAAUCCAGCUACCAUAAUGAU<br>ACAGAAAGGCCCUCGUGCACC	Pull-down assay	Fig. 6	*in vitro* transcription

Supplementary Notes

Supplementary Note 1 | Possible explanations on different binding intensities of the rG4 controls, related to Fig.5d, 5e

We found that the G-scores are positively correlated with the CIRBP binding intensities (Fig. 5d, middle). The RGG motif (arginine-glycine-glycine motif) of CIRBP is vital for its binding to G4 in π-stacking interactions[1]. As the number of rG4 layers increases, the rG4 structure stabilizes and the π-stacking interaction with the RGG motif is strengthened. This is likely the reason why the CIRBP binding intensities were positively correlated with the G-scores. On the other hand, although DHX36 binds to rG4 with the highest specificity, it did not show a positive correlation with either the G-scores or G numbers (Fig. 5d, bottom). Instead, we found that RNAs with a low number of Gs and low number of G4 layers, r(UGGU)8, and r(UGGU)6 had the highest binding intensity to DHX36 (Fig. 5e). The CIRBP or BG4 data did not show these results. In addition to possible steric hindrances between DHX36 and more stabilized rG4 with a high G-score and G number, DHX36 has helicase activity so that it may prefer interacting with single-stranded RNAs that are caused by the unfolding of less-stable rG4 structures. Unlike CIRBP and DHX36, BG4 has a higher positive correlation with the G numbers than the G4 scores (Fig. 5d, top). Therefore, we assume that the principle of binding between BG4 and rG4 may be different from that of CIRBP and DHX36.

Supplementary Note 2 | Enriched sequence logo generation with k-mer analysis and multiple alignment, related to Supplementary Fig. 11

To discover the protein-binding sequence motif, we constructed a method that is based on k-mer analysis and multiple alignment. As a consequence, the pipeline successfully generated the known U1A-binding sequence motif from FOREST datasets. In the case of LIN28A, we confirmed the CSD binding site (GAU) and the ZKD binding site (GGAG) in the enriched 4-mers[2], and these two distinct motifs were likely represented as a single motif logo. Using this pipeline, we also analyzed three rG4-binding proteins (DHX36, CIRBP, BG4) and found that the enriched sequence motif of DHX36 and CIRBP is consecutive Gs, which is a part of the canonical sequence that can form the rG4 structure. Although the domain of BG4 and detailed mechanism of BG4-binding are still unknown,

we found an enrichment of the BG4-binding sequence motif, which is unlike the motifs of DHX36 and CIRBP.

Supplementary Note 3 | A comprehensive comparison of the rG4-specific stabilization, related to Supplementary Fig. 12

To distinguish rG4-forming RNA from cross-reactive RNA, we compared the binding intensities in the presence of potassium ions or lithium ions (Supplementary Fig. 12). Because the ionized diameter of potassium is suitable for stabilizing the layered structure, potassium ions can specifically stabilize rG4. Thus, the probability of rG4 is higher in the presence of potassium ions than of smaller cationic ions. Accordingly, we assumed that the BG4-binding intensity of rG4 would increase in the presence of potassium compared with that in the presence of lithium, and the difference in intensities would reflect rG4-specific stabilization, making it useful to identify rG4. We then calculated the rG4-stabilizing scores (Δnormalized binding intensities) from each quantitative BG4-binding intensity. As expected, all rG4 controls showed nonnegative values, indicating that the probability of rG4 formation was higher in the potassium buffer.

Supplementary Note 4 | Effect on RNP interaction by competition between Watson-Crick base pairs and Hoogsteen base pairs, related to Supplementary Figs.14 and 15

We noticed that although some pre-miRNA loops contain potential rG4 sequences, they do not show high binding intensities (e.g., *hsa-mir-1234*). We reasoned that these differences could be explained by competition between the rG4 and duplex structures, as shown in a previous report[3]. Indeed, secondary structure analysis of the G-tracts revealed that the Watson-Crick base-pair probability is negatively correlated with the BG4-binding intensities (Supplementary Fig. 14).

To further investigate this observation, we performed a multiplex pull-down assay by FOREST in a crowding environment with small molecules (PEG 200 40% w/v), in which the Watson-Crick base pairing is destabilized and the Hoogsteen base pairing is stabilized[4,5], and analyzed whether the BG4 binding intensities change (Supplementary Fig. 15). As a result, we observed a significant enhancement in the binding of the *hsa-mir-1234* loop to BG4 in the presence of PEG 200. Similarly, other pre-miRNAs (e.g., hsa-mir-1224) containing rG4-forming sequences in their stems showed increases in BG4 binding intensities under the molecular crowding environment

with the small molecules. These results indicated that G-tracts forming Watson-Crick base-pairing under the dilute solution condition are transformed into rG4 structures by the molecular crowding effect.

Supplementary Note 5 | Pull-down with cell lysates containing overexpressed and FLAG-tagged proteins, related to Supplementary Fig. 17

To simplify and generalize the protein preparation step in FOREST, we used cell lysates expressing FLAG-tagged RNA-binding proteins (Supplementary Fig. 17a). Using mouse Roquin (RC3H1) as a model protein, we performed FOREST with anti-FLAG antibody-conjugated beads and cell lysates containing FLAG-Roquin to verify the detection of Roquin-binding controls that were included in the RNA structure library, v2. As a result, we confirmed the enrichment of the control RNA motifs with remarkable rankings and scores (Supplementary Fig. 17b). Similarly, several RNA structures in the human 5'UTRs were found with high binding intensities, and the U-R-U triloop motif was confirmed in them (Supplementary Fig. 17c). These results indicate that overexpressed cell lysates allow us to construct a simple system for evaluating FOREST without the need for protein purification or the generation of specific antibodies.

Supplementary Discussion

Future challenge of FOREST

In the future, the RNA structure library in FOREST could be used as a tool to find RNA structure-targeted drugs. In order to screen RNA-binding molecules in a high-throughput manner, current methods often use random RNA sequences by small-molecule microarrays[6]. The RNA structure library could be combined with these techniques, expanding the RNA-ligand interaction landscape with annotation to RNA class or genes. Additionally, the library may be applied to identify other functional RNA elements. For example, it could be used to analyze self-cleaving ribozymes from genomic sequences by combining PAGE purification and barcoded RNA systems[7]. Thus, we believe that FOREST could provide a general platform for studying various RNA structure-function relationships.

Supplementary Methods

Structural context analysis of targeted sequence motifs, related to Supplementary Figs. 7 and 14

Following a previous study[8], we used RNAsubopt provided in the ViennaRNA package[9] with setting parameters (command: RNAsubopt --temp=4 --stochBT=30). Using the input of the secondary structures produced by RNAsubopt, the loop probabilities were calculated by a Python 3.7 custom script. The target sequence motifs were set to “NGCACN” for U1A and “Gn (n≧3)” for BG4. N stands for any nucleotide A, U, G, or C.

RNA biotinylation, related to Supplementary Fig. 10

First, 150 pmol of in vitro transcribed RNA (r(UGGGGU)8, r(GAA)16, hsa-mir-16-1) was biotinylated at the 3’ end with 3 nmol of biotinylated cytidine by T4 RNA ligase using an RNA biotinylation kit (Pierce RNA 3’ End Biotinylation Kit, Thermo Fisher Scientific). Then, the assembled reactions were incubated at 16 °C for 20 hours. The reactions were purified with purification columns (RNA Clean & Concentrator-5, Zymo Research) and eluted with 20 µL of nuclease-free water. Details of the RNA probes are available in Supplementary Table 2.

Enzyme-linked immunosorbent assay (ELISA) of RNA:BG4 interactions, related to Supplementary Fig. 10

Biotinylated RNA (200 nM) in hybridization buffer (10 mM Tris-HCl pH 7.5, 100 mM KCl) was refolded by incubation at 95 °C for 5 min and snap cooled down on ice. Then, 400 nM of BG4 Fab (Absolute Antibody) in binding buffer (10 mM Tris-HCl pH 7.5, 100 mM KCl, 0.4 mg/mL BSA) was sequentially 2-fold diluted 8 times. Next, 25 µL of 200 nM biotinylated RNA (5 pmol) and 25 µL of different concentrations of BG4 antibody (0-10 pmol) were mixed and incubated at 4 °C for 30 min. Neutravidin-immobilized wells (Pierce NeutrAvidin Coated High Capacity Plates, Thermo Fisher Scientific) were washed with TBS-T (TBS pH 7.4 with 0.05% Tween 20) three times. The mixtures were transferred to the wells and incubated at room temperature for 1 hour. The plates were washed with TBS-T six times. Fifty microliters of HRP conjugated anti-6xhistidine antibodies (6x-His Tag Monoclonal Antibody (3D5) HRP, Thermo Fisher Scientific, R931-25) 1000-fold diluted in

blocking buffer (SuperBlock T20 TBS Blocking Buffer, Thermo Fisher Scientific) were added to the wells, which were then incubated at room temperature for 1 hour. The plates were washed with TBS-T six times. Fifty microliters of TMB (1-Step Ultra TMB-ELISA Substrate Solution, Thermo Fisher Scientific) was added to each well, and the wells were then incubated at room temperature for 15 minutes. The reaction was stopped by adding 50 µL of 1.5 M sulfuric acid to each well. The 450 nm absorbance of the reactions was measured with a Microplate reader (Infinite M1000, Tecan). The experiments were performed with three independent samples, and the standard errors of the mean were calculated.

Calculation of Δnormalized BG4-binding intensities (ΔK-Li), related to Supplementary Fig. 12

Two sets of binding data (potassium and lithium conditions) were normalized by quantile normalization using a Julia 1.1 custom script. ΔK-Li was calculated by subtracting the normalized binding intensities under potassium buffer from those under lithium buffer.

Circular dichroism (CD) spectra analysis, related to Supplementary Fig. 13

All RNAs were synthesized and purchased from GeneDesign. RNA samples (1.5 µM) were prepared in 1× K^+ folding buffer or 1× Li^+ folding buffer. All RNA samples were incubated at 95 °C for 5 minutes and 4 °C for at least 5 minutes. Before the analysis, the samples were incubated at room temperature for at least 5 minutes. CD spectra were acquired on a J-720WI (JASCO) with a Peltier temperature controller set to 25 °C. All samples were analyzed within a micro quartz cell with a 1 cm path length. The data were averaged over five scans with subtraction of the buffer baseline. Details of the RNA probes are available in Supplementary Table 2.

Sequence-based rG4 classification, related to Supplementary Fig. 14

We defined four types of rG4 with regular expressions and labeled the 1916 RNAs in RNA structure library, v1, with rG4 subclasses using custom script written in Julia 1.1. The regular expressions are represented as follows. (1) Canonical rG4 was defined as "(G{3,}.{1,7}){3}G{3}" or "(G{4,}.{1,7}){4}G{4}". (2) Long-loop rG4 was defined as "((G{3}.{1,12}){3}.G{3}" or "G{3}.{1,7}G{3}.{13,21}G{3}.{1,7}G{3}". (3-1) Bulge rG4 (large insertion-type) was defined as the combination of three G{3} and a single "GG[^G]{1,7}G" or "G[^G]{1,7}GG". For example,

"(G{3}.{1,9}G{3}.{1,9}G{3}.{1,9}(GG[^G]{1,7}G)" indicates bulge rG4 with a large insertion at the last G-tract. (3-2) Bulge rG4 was defined as "((G{3}|GG[^G]|G[^G]GG).{1,9}){3}(G{3}|GG[^G]G|G[^G]GG)". (4) 2-Tetrad was defined as "(G{2}.{1,9}){3}G{2}". If an RNA was categorized into multiple classes, the RNA was assigned to the class that had higher stability (canonical rG4 > long loop > bulge > 2-tetrad)[3], Then we calculated the average BG4-binding intensities of each population.

RNA pull-down with BG4 protein and polyethylene glycol 200, related to Supplementary Fig. 15

The RNA structure library was prepared in 1× PKN folding buffer (20 mM phosphate buffer pH 7.0, 140 mM KCl, 10 mM NaCl) with or without 40% w/v PEG 200 (Nacalai tesque). For folding, RNA was heated at 95 °C and cooled to 4 °C at −6 °C/sec on a ProFlex Thermal Cycler (Thermo Fisher Scientific). For this purpose, 100 pmol of BG4, 20 µL of TALON magnetic beads (Clontech), and 1 µg of the refolded RNA structure library were mixed in 1 mL of 1× PKN binding buffer (20 mM phosphate buffer pH 7.0, 140 mM KCl, 10 mM NaCl and 0.1 µg/µL BSA) with or without 40% w/v PEG 200. A mixture containing no protein was also prepared as a control. The mixture was incubated on a rotator at 4 °C for 60 minutes and washed three times with 1× BG4 protein-binding buffer. Then, 200 µL of elution buffer was added to the magnetic beads, and the mixture was heated at 95 °C for 3 minutes. The RNA was collected from the supernatant by removing the magnetic beads. The RNA structure library in the mixture was extracted with phenol and chloroform with ethanol precipitation for purification.

Preparation of cell lysates containing overexpressed FLAG-proteins, related to Supplementary Fig. 17

293FT cells (3 × 10^6 cells) were seeded in a 10-cm cell culture dish and cultured for 24 hours before transfection. Fifteen micrograms of pFLAG-Roquin (a gift from Dr. Osamu Takeuchi) was transfected into the cells by Lipofectamine 3000 (Thermo Fisher Scientific, #L3000008) following the manufacturer's instructions. As a mock transfection control, the cells were treated with Lipofectamine 3000 alone. After 24 hours, the treated cells were collected by centrifugation. The cell pellets were washed with ice-cold PBS and lysed by incubation for 30 min on ice with intermittent

vortex mixing in 1 ml of Cell Lysis Buffer (Invitrogen, #FNN0021) with 1× cOmplete Protease Inhibitor Cocktail (Roche, #04693116001) and 1 mM phenylmethylsulfonyl fluoride (Thermo Fisher Scientific, #36978). The cell lysates were centrifuged at 13,000 rpm for 10 min at 4°C, and the resulting supernatants were collected. The cell lysates were stored at –80°C until use.

Supplementary References

1. Huang, Z.-L. *et al.* Identification of G-Quadruplex-Binding Protein from the Exploration of RGG Motif/G-Quadruplex Interactions. *J. Am. Chem. Soc.* **140**, 17945–17955 (2018).
2. Ustianenko, D. *et al.* LIN28 Selectively Modulates a Subclass of Let-7 MicroRNAs. *Mol. Cell* **71**, 271–283.e5 (2018).
3. Kwok, C. K., Marsico, G., Sahakyan, A. B., Chambers, V. S. & Balasubramanian, S. rG4-seq reveals widespread formation of G-quadruplex structures in the human transcriptome. *Nat. Methods* **13**, 841–844 (2016).
4. Nakano, S.-I., Miyoshi, D. & Sugimoto, N. Effects of molecular crowding on the structures, interactions, and functions of nucleic acids. *Chem. Rev.* **114**, 2733–2758 (2014).
5. Matsumoto, S., Tateishi-Karimata, H., Takahashi, S., Ohyama, T. & Sugimoto, N. Effect of Molecular Crowding on the Stability of RNA G-Quadruplexes with Various Numbers of Quartets and Lengths of Loops. *Biochemistry* **59**, 2640–2649 (2020).
6. Connelly, C. M., Abulwerdi, F. A. & Schneekloth, J. S., Jr. Discovery of RNA Binding Small Molecules Using Small Molecule Microarrays. *Methods Mol. Biol.* **1518**, 157–175 (2017).
7. Nomura, Y., Chien, H.-C. & Yokobayashi, Y. Direct screening for ribozyme activity in mammalian cells. *Chem. Commun.* **53**, 12540–12543 (2017).
8. Dominguez, D. *et al.* Sequence, Structure, and Context Preferences of Human RNA Binding Proteins. *Mol. Cell* **70**, 854–867.e9 (2018).
9. Lorenz, R. *et al.* ViennaRNA Package 2.0. *Algorithms Mol. Biol.* **6**, 26 (2011).

Supplementary Data (zip)

Supplementary Data 1: Reporter mRNA sequences related to Fig.7 (xlsx).

Supplementary Data 2: RNA structure library version 1 (csv).

Supplementary Data 3: RNA structure library version 2 (csv).

Supplementary Data 4: Hybridization test (csv).

Supplementary Data 5: FOREST data of U1A (csv).

Supplementary Data 6: FOREST data of LIN28A (csv).

Supplementary Data 7: FOREST data of BG4 (csv).

Supplementary Data 8: FOREST data of CIRBP (csv).

Supplementary Data 9: FOREST data of DHX36 (csv).

Supplementary Data 10: FOREST data of EIF3-complex (csv).

Supplementary Data 11: FOREST data of BG4 under molecular crowding condition (csv).

Supplementary Data 12: FOREST data of FLAG-Roquin (csv).

Supplementary Data 13: Predicted RNA structures of the functional regions for NEAT1 sublibrary (txt).

Supplementary Data 5–12 include s.d., s.e.m., and coefficient of variation among multiple barcodes assigned to each RNA.
