# Supplementary material for: RNA structure-wide discovery of functional interactions with multiplexed RNA motif library
Source: Nat Commun. 2020 Dec 8;11:6275. doi: 10.1038/s41467-020-19699-5 (PMC7723054; doi:10.1038/s41467-020-19699-5)
Supplement: Supplementary file 2 — Reporting Summary [file 41467_2020_19699_MOESM2_ESM.pdf]

nature research

Corresponding author(s): Hirohide Saito, Shunnichi Kashida

Last updated by author(s): Sep 29, 2020

Reporting Summary

Nature Research wishes to improve the reproducibility of the work that we publish. This form provides structure for consistency and transparency in reporting. For further information on Nature Research policies, see Authors & Referees and the Editorial Policy Checklist.

Statistics

For all statistical analyses, confirm that the following items are present in the figure legend, table legend, main text, or Methods section.

n/a	Confirmed	
☐	☒	The exact sample size (*n*) for each experimental group/condition, given as a discrete number and unit of measurement
☐	☒	A statement on whether measurements were taken from distinct samples or whether the same sample was measured repeatedly
☐	☒	The statistical test(s) used AND whether they are one- or two-sided<br>*Only common tests should be described solely by name; describe more complex techniques in the Methods section.*
☒	☐	A description of all covariates tested
☐	☒	A description of any assumptions or corrections, such as tests of normality and adjustment for multiple comparisons
☐	☒	A full description of the statistical parameters including central tendency (e.g. means) or other basic estimates (e.g. regression coefficient) AND variation (e.g. standard deviation) or associated estimates of uncertainty (e.g. confidence intervals)
☐	☒	For null hypothesis testing, the test statistic (e.g. *F*, *t*, *r*) with confidence intervals, effect sizes, degrees of freedom and *P* value noted<br>*Give P values as exact values whenever suitable.*
☒	☐	For Bayesian analysis, information on the choice of priors and Markov chain Monte Carlo settings
☒	☐	For hierarchical and complex designs, identification of the appropriate level for tests and full reporting of outcomes
☐	☒	Estimates of effect sizes (e.g. Cohen's *d*, Pearson's *r*), indicating how they were calculated

Our web collection on statistics for biologists contains articles on many of the points above.

Software and code

Policy information about availability of computer code

Data collection	Centroid fold version 0.0.15<br>ParasoR version 1.1.0<br>RemovePseudoKnots and ct2dot in RNAstructure website [https://rna.urmc.rochester.edu/RNAstructureWeb/]
Data analysis	Forna website [https://rna.urmc.rochester.edu/RNAstructureWeb/]<br>RNAcofold and RNAsubopt in ViennaRNA package 2.4.<br><br>QGRS Mapper website [http://bioinformatics.ramapo.edu/QGRS/help_search.php]<br>Clustal Omega website [https://www.ebi.ac.uk/Tools/msa/clustalo/]<br>MEME website [http://meme-suite.org/tools/meme]<br>Weblogo 3 website [http://weblogo.threeplusone.com/create.cgi] for generating a motif logo in Fig.6a<br><br>The custom scripts for the motif extraction of the terminal motifs, designing the RNA structure library, and generation of the microarray ordering template are available in the Github page with the instruction to order as products (https://github.com/KRK13/FOREST2020/).

For manuscripts utilizing custom algorithms or software that are central to the research but not yet described in published literature, software must be made available to editors/reviewers. We strongly encourage code deposition in a community repository (e.g. GitHub). See the Nature Research guidelines for submitting code & software for further information.

Data

Policy information about availability of data

All manuscripts must include a data availability statement. This statement should provide the following information, where applicable:

- Accession codes, unique identifiers, or web links for publicly available datasets
- A list of figures that have associated raw data
- A description of any restrictions on data availability

We declare that all data supporting the results in this study are available within the paper and its Supplementary Information.
Raw data are available from the corresponding author on request.

To design RNA structure library version 1, we extracted pre-miRNA loops from miRBase ver.21 [http://www.mirbase.org/].
Additionally, we extracted terminal motifs of NEAT1 from the RNA secondary structures predicted by ParasoR (Supplementary Data 13) and PARIS dataset of HeLa cell [Lu, Z. et al. RNA Duplex Map in Living Cells Reveals Higher-Order Transcriptome Structure. Cell 165, 1267–1279 (2016)].

To design RNA structure library version 2, we extracted terminal motifs from UTRdb [http://utrdb.ba.itb.cnr.it] and SHAPE-MaP dataset of HIV-1 genome [Siegfried, N. A., Busan, S., Rice, G. M., Nelson, J. A. E. & Weeks, K. M. RNA motif discovery by SHAPE and mutational profiling (SHAPE-MaP). Nat. Methods 11, 959–965 (2014)].

To design a barcode microarray, we used the datasets of barcodes for the hybridization of nucleic acid [Xu, Q., Schlabach, M. R., Hannon, G. J. & Elledge, S. J. Design of 240,000 orthogonal 25mer DNA barcode probes. Proc. Natl. Acad. Sci. U. S. A. 106, 2289–2294 (2009)].

Field-specific reporting

Please select the one below that is the best fit for your research. If you are not sure, read the appropriate sections before making your selection.

☒ Life sciences ☐ Behavioural & social sciences ☐ Ecological, evolutionary & environmental sciences

For a reference copy of the document with all sections, see nature.com/documents/nr-reporting-summary-flat.pdf

Life sciences study design

All studies must disclose on these points even when the disclosure is negative.

Sample size

We did not use statistical methods to determine sample size. The entire population was used for the initial data analysis of FOREST. Further data analysis of FOREST was performed using all RNA probes that met the criteria for the binding intensities and the sequence features.

Data exclusions

We omitted the binding intensity of the hsa-let-7f-2 loop from the calculation shown in Fig.4e. EMSA probed that the hsa-let-7f-2 loop does not strongly interact with LIN28A, unlike other class-2 let-7 loops. The exclusion criteria were pre-established because the analysis in Fig 4e was designed for evaluation using LIN28A-binding RNA probes, not low-affinity RNA probes.

Replication

EMSA experiments were performed three times; the band intensities were quantified; the same results were consistently obtained.
ELISA was performed three times; the same results were consistently obtained.
Immunoblot was performed at least twice; the same results were consistently obtained.
Biochemical and cell experiments were performed with three biological replicates, each with two technical replicates. The same results were consistently obtained throughout replicated experiments.

In FOREST, all DNA barcodes were immobilized to at least two different spots in the same array (n=5; v1, n=2; v2).
All RNA probes in RNA structure libraries were replicated, as each structure was assigned to different RNA barcodes (n=5; v1, n=3; v2).
FOREST assays were performed mostly two times as follows: Hybridization test; n=2, U1A; n=2, LIN28A; n=2, BG4 K+; n=2, CIRBP; n=2, DHX36; n=2, EIF3 complex; n=2. The similar results were consistently obtained throughout replicated experiments.
The preliminary and related experiments repeatedly confirmed the robustness of FOREST with BG4 and Roquin. Therefore these experiments were tested once (BG4 Li+; n=1, BG4 PEG; n=1, Roquin; n=1.). As a result, positive spike-in controls were correctly evaluated, showing a similar trend in the related experiments.

Randomization

Randomization was not necessary for the multiplexed assay of FOREST because all RNA probes in an RNA structure library were randomly distributed and simultaneously evaluated in a single tube. All RNA probes were used in both groups, with or without the target protein.

Randomization was not necessary for data analysis of FOREST and the related validation experiments because all subgroups were objectively determined by quantitative analysis on the binding intensities and RNA sequences.

Blinding

Blinding was not relevant for this study as experiments were assessed quantitatively based on objective.

Reporting for specific materials, systems and methods

We require information from authors about some types of materials, experimental systems and methods used in many studies. Here, indicate whether each material, system or method listed is relevant to your study. If you are not sure if a list item applies to your research, read the appropriate section before selecting a response.

Materials & experimental systems

n/a	Involved in the study	
☐	☒	Antibodies
☐	☒	Eukaryotic cell lines
☒	☐	Palaeontology
☒	☐	Animals and other organisms
☒	☐	Human research participants
☒	☐	Clinical data

Methods

n/a	Involved in the study	
☒	☐	ChIP-seq
☒	☐	Flow cytometry
☒	☐	MRI-based neuroimaging

Antibodies

Antibodies used

Rabbit anti-eIF3B/EIF3S9 antibody (Bethyl Laboratories, A301-761A) diluted 1:20 for immunodetection.

Rabbit anti-RPS19 antibody (Bethyl Laboratories, A304-002A) diluted 1:50 for immunodetection.

Beta-Actin (D6A8) Rabbit mAb (Cell Signaling Technology, 8457S) diluted 1:50 for immunodetection.

Anti-DNA/RNA G-quadruplex (and related small molecules) [BG4], Mouse Fab fragment, His-Tagged, Lambda (Absolute antibodies, Ab00174-1.6)

Monoclonal ANTI-FLAG® M2 antibody (Sigma-Aldrich, F1804)

6x-His Tag Monoclonal antibody (3D5) HRP (Thermo Scientific, R931-25)

HRP-conjugated anti-rabbit (ProteinSimple, 042-206)

Validation

A304-002A, A301-761A: Lee, A. S. Y., Kranzusch, P. J. & Cate, J. H. D. eIF3 targets cell-proliferation messenger RNAs for translational activation or repression. Nature 522, 111–114 (2015).

8457S: https://www.cellsignal.jp/products/primary-antibodies/b-actin-d6a8-rabbit-mab/8457

Ab00174-1.6: https://absoluteantibody.com/product/anti-dnarna-g-quadruplex-and-related-small-molecules-bg4/Ab00174-1.6_Mouse_Fab_fragment/

F1804: Anantharaman, A. et al. ADAR2 regulates RNA stability by modifying access of decay-promoting RNA-binding proteins. Nucleic Acids Res. 45, 4189–4201 (2017)

R931-25: Deng, L. et al. Heterosubtypic influenza protection elicited by double-layered polypeptide nanoparticles in mice. Proc. Natl. Acad. Sci. U. S. A. 115, E7758–E7767 (2018)

Eukaryotic cell lines

Policy information about cell lines

Cell line source(s)	293FT Cell Line (R70007) was obtained from Thermo Fisher Scientific.
Authentication	None of the lines have been authenticated.
Mycoplasma contamination	293FT Cell Line was tested for mycoplasma contamination and proven negative.
Commonly misidentified lines (See ICLAC register)	No commonly misidentified cell lines were used in this study
