# Supplementary material for: RNA structure-wide discovery of functional interactions with multiplexed RNA motif library
Source: Nat Commun. 2020 Dec 8;11:6275. doi: 10.1038/s41467-020-19699-5 (PMC7723054; doi:10.1038/s41467-020-19699-5)
Supplement: Supplementary file 3 — Description of Additional Supplementary Files [file 41467_2020_19699_MOESM3_ESM.pdf]

Description of Additional Supplementary Files

File Name: Supplementary Data 1
Description: Reporter mRNA sequences related to Fig.7 (xlsx).

File Name: Supplementary Data 2
Description: RNA structure library version 1 (csv).

File Name: Supplementary Data 3
Description: RNA structure library version 2 (csv).

File Name: Supplementary Data 4
Description: Hybridization test (csv).

File Name: Supplementary Data 5
Description: FOREST data of U1A (csv).

File Name: Supplementary Data 6
Description: FOREST data of LIN28A (csv).

File Name: Supplementary Data 7
Description: FOREST data of BG4 (csv).

File Name: Supplementary Data 8
Description: FOREST data of CIRBP (csv).

File Name: Supplementary Data 9
Description: FOREST data of DHX36 (csv).

File Name: Supplementary Data 10
Description: FOREST data of EIF3-complex (csv).

File Name: Supplementary Data 11
Description: FOREST data of BG4 under molecular crowding condition (csv).

File Name: Supplementary Data 12
Description: FOREST data of FLAG-Roquin (csv).

File Name: Supplementary Data 13
Description: Predicted RNA structures of the functional regions for NEAT1 sublibrary (txt).
